# COVID-19 kit distribution in six Latin American countries: bureaucratic autonomy, populism, and evidence-based policymaking

**DOI:** 10.1093/heapol/czag069

**Published:** 2026-05-20

**Authors:** José Antonio Requejo Domínguez, Kevin Croke, Veronika J Wirtz

**Affiliations:** Department of Health Policy, London School of Economics and Political Science (LSE), Houghton Street, London WC2A 2AE, United Kingdom; Department of Global Health, Boston University School of Public Health, 715 Albany St, Boston, MA 02118, United States; Department of Global Health and Population, Harvard T.H. Chan School of Public Health, 677 Huntington Ave, Boston, MA 02115, United States; Department of Global Health, Boston University School of Public Health, 715 Albany St, Boston, MA 02118, United States

**Keywords:** COVID-19 kits, populism, bureaucratic autonomy, National Medicines Regulatory Agencies, Health Technology Assessment Organizations

## Abstract

During the COVID-19 pandemic, governments varied widely in the extent to which policy decisions aligned with scientific evidence. We examine decision-making regarding the public distribution of COVID-19 kits containing ivermectin in six Latin American countries in 2020 and 2021, despite international scientific authorities advising against using these medicines outside of clinical trials. We focus on institutions, ideology, and interests. Theories emphasising the centrality of institutions predict that autonomous scientific institutions are more likely to resist political pressure to adopt ineffective interventions. Theories focused on ideology suggest that populist leaders may favour highly visible policy responses, including ineffective treatments, even when these diverge from expert advice. A third approach emphasises the relative power of interest groups favouring or opposing kit distribution. Using the comparative case study method, we analyse policy processes across Guatemala, Peru, Mexico, Costa Rica, Colombia, and Argentina. We find that neither bureaucratic autonomy, nor populism, nor interests alone explains policy variation. Instead, the findings are consistent with a conditional relationship in which populist leaders are more likely to favour kit distribution, but autonomous National Medicines Regulatory Agencies appear to moderate the influence of populist political pressures. Countries with non-populist leaders but low institutional autonomy were also likely to distribute kits if powerful political actors favoured such policies. These findings suggest that institutional design and political context jointly shape the capacity for evidence-informed decision-making during public health emergencies. Strengthening the autonomy of regulatory agencies may help support evidence-based evaluation of emerging health technologies during future crises.

Key messages

- Governments varied in the degree to which they made evidence-based policies during the COVID-19 pandemic.
- Using comparative case study methods, we examine the policy process by which six countries in Latin America decided whether to distribute COVID-19 kits containing treatments that scientific authorities had judged to be ineffective or potentially harmful, such as ivermectin.
- We focus on institutions, ideology, and interests.
- We find that bureaucratic autonomy can moderate the impact of populism on policymaking processes.
- Strengthened autonomy of Latin American regulatory agencies could promote effective decision-making during health crises.

## Introduction

The COVID-19 pandemic had a significant impact on Latin America (OECD 2020). The high prevalence of chronic diseases, the lack of universal access to quality healthcare services, and broader economic inequalities meant that the region was ill-prepared for the sudden increase in COVID-19 infections (OECD 2020, Pan American Health Organization 2023). COVID-19 overwhelmed the health systems of several countries, causing a large number of cases and deaths (Etienne 2021).

Some countries distributed kits containing medicines meant for treating COVID-19 that scientific authorities and leading international organizations had neither validated nor deemed effective. These kits contained medicines such as ivermectin, azithromycin, hydroxychloroquine, loratadine, and prednisone, as well as vitamins (Salud con lupa 2020). Local or national governments in at least seven countries (Mexico, Guatemala, Brazil, Peru, Panama, Honduras, and El Salvador), representing almost 40% of the population of North, Central, and South America, decided to distribute COVID-19 kits (Camhaji Galarraga Gortázar and Santaeulalia 2022).

Notably, these kits could pose harm to the population. For instance, common adverse reactions to ivermectin include headache, pruritus, muscle pain, cough, dyspnoea, nausea, vomiting, diarrhoea, and postural hypotension (Campillo et al. 2021). Rare adverse reactions include complex inflammatory responses called Mazzotti reactions (Campillo et al. 2021). Similarly, the use of azithromycin as an antibiotic in the absence of diagnosis can increase antimicrobial resistance (Murray et al. 2022). In some countries, the kits contained prednisone, a corticosteroid, which suppresses the immune system, and can cause indigestion, heartburn, and fluid retention among other side effects (U.S. Food and Drug Administration 2024). Loratadine, an antihistamine used for the treatment of allergies (U.S. Food and Drug Administration 2000), was also part of the COVID-19 kits in several countries. Loratadine also has side effects and was not recommended at any point during the COVID-19 pandemic as an effective treatment. Table 1 shows the contents of different kits across five Latin American countries.

**Table 1:** COVID-19 kit content in five Latin American countries.

	El Salvador	Guatemala^a^	Brazil	Mexico^a^	Peru^a^
Acetaminophen	x	x			x
Acetylsalicylic acid	x	x	x		
Azithromycin	x	x		x	x
Hydroxychloroquine			x		
Ivermectin	x	x	x	x	x
Loratadine	x	x			
Prednisone			x		
Vitamin C, D, and zinc	x	x			

^a^Countries included in this study.

Studies analysing policy decision-making during the pandemic have found wide variation across Latin American countries in the degree to which they used evidence in their policy decision-making (Knaul et al. 2021). Advocates of COVID-19 kits justified their use on the grounds that responding to the crisis was more important than waiting for rigorous evidence or relying on scientific experts. Scientific institutions largely advised against using these medicines. Yet scientists across the world faced pressure and at times clashed with political leaders (Colman et al. 2021), and it was not clear *ex ante* whether interest groups and institutions representing evidence-informed policy would see their recommendations adopted in the face of opposition.

Relatedly, populism surged during the pandemic. While there is an emerging literature on links between populism and health policy (Falkenbach and Greer 2018), the field of public health has not sufficiently examined how populism shapes the political and institutional conditions for evidence-based policymaking, especially during crises (Stanford et al. 2022). In general, there is a lack of research on the role of institutions and the broader political context in fostering the conditions for evidence-based policymaking in the region. We address this gap by assessing the relative role of institutions, ideology, and interests in policymaking related to COVID-19 kit distribution. Institutions, ideology, ideas, and interests are four explanatory variables often used in political science to analyze health policy processes (Hall 1997, Fox and Reich 2015). We consider three theoretical concepts related to evidence-based decision-making during the pandemic which map onto this framework: ‘bureaucratic autonomy’ (institutions), ‘populism’ (ideology), and the relative power of groups favouring or opposing kit distribution in the policymaking process (interests).

In this paper, we investigate why some Latin American governments decided to distribute COVID-19 kits containing ivermectin, examining the extent to which bureaucratic autonomy, populism, and interest groups explain variation in evidence-based decision-making across six countries. We focus specifically on COVID-19 kits containing ivermectin, as it was ubiquitous in countries that distributed such kits.

## Conceptual framework

### Role of bureaucratic autonomy

The concept of bureaucratic autonomy is used by political scientists and public administration scholars as part of the broader study of state capacity. Bureaucratic autonomy is the ability of executive agencies to use their discretionary authority to implement policies made by political principals, as well as to make policy according to their wishes when mandates are ambiguous, incomplete, corrupt, or contrary to their perception of national interest (Bersch and Fukuyama 2023). Bureaucratic autonomy is not an absolute good. In democratic societies, civil servants are ultimately responsible to their legitimately chosen political leaders. However, public administration scholars have identified components of operational autonomy as key drivers of state capacity, in particular when governments must make policy in specialized areas in which technical expertise is needed (Evans 1995). As such, it is a factor that supports the use of evidence in policymaking.

Bureaucratic autonomy has been studied in the context of National Medicines Regulatory Agencies (NMRAs) and Health Technology Assessment Organizations (HTAOs), where it is central to their ability to effectively regulate the pharmaceutical industry and provide independent, evidence-based advice to governments (Carpenter 2010) (see box 1). Nevertheless, the presence or absence of bureaucratic autonomy in NMRAs and its relationship to evidence-based decision-making during COVID-19 in low- and middle-income countries remains poorly documented, despite the central role of these agencies in providing clinical guidance in pharmacological treatments during the pandemic (Pan American Health Organization 2021). During the COVID-19 pandemic, several Latin American governments formed technical advisory boards composed of multiple technical institutions, such as NMRAs and HTAOs, to appraise evidence and advise policymakers on the best way to tackle the crisis (Pan American Health Organization 2021).

Box 1 **Description of the responsibilities of NMRAs and HTAOs**

**Table:** 

**National Medicines Regulatory Agencies (NMRAs)** NMRAs are government bodies responsible for ensuring that pharmaceutical products meet safety, efficacy, and quality standards before they are made available to the public. During the pandemic, the role of NMRAs in providing clinical guidance on pharmacological treatments became vital (World Health Organization 2023a). NMRAs rapidly assessed newly available national and international evidence on potential COVID-19 treatments and continuously updated their recommendations accordingly.
**Health Technology Assessment Organizations (HTAOs)** HTAOs systematically evaluate the clinical effectiveness, cost-effectiveness, and broader implications of health technologies to inform policy and coverage decisions (World Health Organization 2025). During the pandemic, HTAOs had to appraise emerging evidence on novel treatments under significant time pressure and uncertainty, providing governments with recommendations on which interventions should or should not be adopted.

In theory, the degree of bureaucratic autonomy of NMRAs and HTAOs determines the extent to which these agencies can base their decisions on scientific evidence and act to protect public health without conflict of interest. The financial and operational independence of NMRAs and HTAOs from elected officials can mitigate conflicts of interest and insulate them from political pressure. By contrast, NMRAs and HTAOs that lack autonomy risk making decisions that align with the preferences of political leaders, even when these diverge from international guidelines or their professional evaluation of existing evidence.

### Populism

Scholars often study the role of ideology in shaping health policy choices (Esping-Andersen 1998, Lynch 2020). In the context of the COVID-19 pandemic, one political ideology that has been identified as relevant to policy is populism. Populism is a political movement that has gained strength globally over the past decade, including in Latin America, the United States, the United Kingdom, India, Turkey, and the Philippines (Campos and Casas 2021, Singh 2021). Populist movements share features, including symbolic narratives in which ‘the people’ are perceived as threatened by an elite group, while also favouring a majority ethnic or religious group (Mudde and Kaltwasser 2013, Fukuyama 2018). It is also characterized by popular, simplistic, short-term-oriented policies that may be unsustainable. Another noted feature of populism is ‘the ideology that considers society to be ultimately separated into two homogeneous and antagonistic groups, ‘the pure people’ versus ‘the corrupt elite’’, which can be extended to include scientific institutions, leading populist leaders to downplay or contradict technocratic or scientific policy advice (Mudde 2004).

In line with this, researchers have argued that populist leaders were more likely to ignore scientific advice or undermine scientific institutions during the pandemic (e.g. President Bolsonaro in Brazil and President Trump in the USA) (Knaul et al. 2021). During past pandemics and epidemics, leaders who employed populist rhetoric promoted harmful and ineffective interventions for political gain, altering the social narrative, influencing health policy, and fuelling antagonism towards academic and scientific elites (Lasco 2020, Lasco and Gregory Yu 2022). Examples include the HIV epidemic in the 2000s and the Ebola outbreak in 2014.

Recent scholarship has shown that rising populism is not only an electoral or discursive phenomenon but also a significant institutional force reshaping the administrative capacities of democratic states. Populist executives often challenge or undermine core bureaucratic values such as expertise, impartiality, meritocracy, and proceduralism, which were once assumed to be stable pillars of consolidated democracies. Studies demonstrate that in several contexts, the administrative insulation traditionally protecting regulatory agencies from political interference has been eroded or even dismantled under populist governments, particularly in policy areas with high symbolic or distributive appeal (Bauer and Becker 2020, Peters and Pierre 2022). This emerging body of work highlights that bureaucratic autonomy cannot be analytically separated from its political environment: populism may actively constrain, politicize, or circumvent autonomous agencies, especially in times of crisis when scientific advice becomes politically consequential.

### Interests

A third approach emphasises the interests of the relevant stakeholders involved in policy debates. Interest group analysis is widely conducted in applied health policy research. For example, Reich and Campos Rivera (2024) propose applied political economy analyses that assess the power and position of key stakeholders inside and outside government when evaluating major health policy reforms. Using this approach, medical associations are often considered key stakeholders in major health policy decisions, as are government health agencies, as well as the private sector, including insurance companies, pharmaceutical firms, and other private health providers. Our analysis in this domain focuses on the relatively small set of institutions and actors, largely in the public sector and political sphere, who were involved in decisions about whether or not to distribute COVID-19 kits.

This comparative, retrospective study aims to examine the decision to distribute COVID-19 kits across six Latin American countries, considering interest-group conflict as well as two factors that previous research has suggested affect the role of evidence in the policymaking process: ‘bureaucratic autonomy’ and ‘populism’. We hypothesized that countries that had previously granted substantial autonomy to NMRAs from political leaders, allowing regulatory institutions to operate on a technocratic basis, would respect scientific advice and, accordingly, limit the distribution of COVID-19 kits. We also hypothesized that in countries with a populist government, political leaders would be more likely to ignore the advice of scientific institutions, including NMRAs and HTAOs, and would be correspondingly more likely to distribute COVID-19 kits.

## Methods

We adopt the comparative case method from political science to analyse the decision-making process surrounding the distribution of COVID-19 kits across six Latin American countries. Following contemporary guidance on comparative case research design (Gerring 2019) and case selection strategies (Mahoney and Goertz 2006, Seawright and Gerring 2008), we selected cases that share important contextual similarities, while varying on our key explanatory variables. This similar-systems design allows us to analyse institutional dynamics and political factors shaping policy decisions in a cross-national context, and to identify plausible mechanisms linking bureaucratic autonomy and populism to policy outcomes.

We proceed as follows. (1) Case selection: Selection of the six countries and review of existing literature to identify relevant institutions and decision-making processes within each country. (2) Identification of independent and dependent variables: We assessed the following independent variables: (i) autonomy of the NMRA and HTAO in each country, (ii) degree of populism, and (iii) the interests and positions of major stakeholders in the COVID-19 kit policy debates. The dependent variable is a dichotomous outcome indicating whether each country distributed COVID-19 kits. (3) Analysis of causal mechanisms: We then classify the institutions involved and their expected role in the decision, given their mandate. This allows us to examine how the policy process evolved and to explore potential causal mechanisms that contribute to observed outcomes via the independent variables. (4) Cross-case comparison: We synthesise the cross-case evidence and analyse common patterns.

Finally, we interviewed local decision-makers to corroborate some of the findings of our analysis.

### Phase one: case selection

Our study sample includes six Latin American countries: Guatemala, Colombia, Costa Rica, Argentina, Peru, and Mexico. These cases share important contextual similarities. All are middle-income countries in Latin America, exposed to similar pandemic waves and operating under comparable international guidance from the World Health Organization (WHO) and the Pan American Health Organization (PAHO). As such, this is a ‘most similar systems’ design (Lijphart 1971, Bennett and Elman 2006, Mahoney and Goertz 2006, Mahoney 2007). There is a well-established literature that uses case studies of Latin American countries as a lens for comparative health system and public policy analysis (Kaufman and Nelson 2004, Weyland 2008).

Within this similar regional, cultural, political, and epidemiological context, these six cases display variation in two key explanatory variables, bureaucratic autonomy and populism, and in the outcome of interest: i.e. the decision of national or subnational authorities to distribute COVID-19 kits containing ineffective and potentially harmful treatments such as ivermectin. In addition, these six countries had differing stakeholder alignments for and against kit distribution.

While comparative case designs are unable to generate counterfactual scenarios, the objective of the study is not causal estimation but rather theory development and the identification of plausible mechanisms through which bureaucratic autonomy moderates the dynamics of populism in health policy-making during crises. These methods are well-suited for process tracing and identification of causal mechanisms (George and Bennett 2005).

#### Document search

Using PubMed, Google Scholar, Nexis Uni, and Google General Search, we conducted a literature review of research articles on policymaking during the pandemic across the six-country sample. The objective was to identify institutions involved in decisions related to public health measures during the pandemic. We included newspaper articles, online news articles, academic publications, and government press releases, focusing on the leading institutions responsible for policy decision-making during the pandemic, in either English or Spanish. Keywords used in the search for English-language publications included ‘Latin America’ and ‘COVID-19 kits’. Other researchers have focused on the impact of medications such as hydroxychloroquine and the negative health consequences associated with its distribution, but this goes beyond the scope of this research paper. Thus, we excluded articles focusing on other medications, such as hydroxychloroquine or azithromycin, articles in languages other than English or Spanish, and articles outside the established dates. Furthermore, we searched for COVID-19 recommendations in the PAHO’s ‘Technical documents and research evidence on COVID-19’, where we identified a monthly systematic review report from April to September 2020, with up-to-date recommendations on the use of ivermectin and other drugs.

### Phase two: identification of independent and dependent variables

#### Dependent variable

The main outcome of interest is whether national or major subnational authorities implemented a policy to distribute COVID-19 kits containing ivermectin to the public during 2020 and 2021.

#### Evaluation of institutional autonomy

We constructed a 3D institutional autonomy index based on the concepts of ‘structural autonomy’ and ‘financial autonomy’ in Verhoest et al.’s (2004) operationalization of bureaucratic autonomy. These dimensions are widely recognised as core components of regulatory agency independence (Gilardi 2002, Hanretty and Koop 2012). Using the documents gathered in phase 1, the official websites of each country’s government and the website and publications of the PAHO, the index evaluated three distinctive elements: (1) whether the NMRA was part of the Ministry of Health, (2) which institution or individual appoints the leader of such an institution, and (3) budgetary autonomy (i.e. independence of the budget from the Ministry of Health). We assessed institutional autonomy at the time of the decision to distribute COVID-19 kits (2020–2021), capturing the formal institutional structure in place.

This index is intended as an operationalization of formal institutional autonomy rather than a comprehensive measure of bureaucratic capacity. Coding decisions were based on publicly available and organizational documents. We confirmed the information with local experts. Where information was ambiguous, we cross-checked from official government websites and secondary literature.

Institutions in each country were assigned to one of two categories: NMRA or HTAO. For instance, for Mexico, we categorized the Federal Commission for the Protection of Health Risks (COFEPRIS) as the NMRA. If all three elements of autonomy are present, the institution receives a high autonomy index score (=3); two points correspond to medium autonomy, and one point to low autonomy. Table 2 provides further details. We hypothesised that the higher the institution’s bureaucratic autonomy, the more likely the organization was to follow evidence-based policies. For example, Argentina’s NMRA, the National Food, Drug, and Medical Technology Administration (ANMAT), is not part of the Ministry of Health, has its own director appointed by the President, and has financial autonomy. Hence, the level of autonomy in our index is high.

**Table 2:** NMRA budget allocation and authority to appoint agency leader.

Country	National Medicines Regulatory Agency	Part of the Ministry of Health	Who appoints the head of the agency?	Is the budget independent of the Ministry of Health?	Degree of autonomy
**Argentina^a^**	ANMAT—National Administration of Drugs, Food and Medical Technology	No	President of the Republic	Yes. The National Budget Office of the Ministry of Finance publishes detailed information regarding the budget allocated by law and budget execution.	Maximum (3)
**Colombia^a^**	Invima—National Institute for the Surveillance of Drugs and Food	No	President of the Republic	Yes. Resources allocated from the National budget.	Maximum (3)
**Mexico**	COFEPRIS—Federal Commission for Protection against Health Risks	Yes	President of the Republic instructs the Secretary of Health for appointment	No.	Medium (2)
**Costa Rica**	Department of Regulation of Products of Sanitary Interest (DRPIS)—Ministerio de Salud, Regulación de Productos de Interés Sanitario Department of Pharmacoepidemiology^b^	Yes	Minister of Health Director of the Costa Rican Social Security Fund (CCSS)^b^	No.	Medium (2)
**Guatemala**	Department of Regulation and Control of Pharmaceutical Products	Yes	Appointed by competent authorities of the Ministry of Public Health and Social Assistance (MSPAS)	No. Resources allocated from the federal government budget to the Ministry of Public Health and Social Assistance, who assigns a part to the Department.	Minimum (1)
**Peru**	DIGEMID—General Directorate of Medicines, Supplies and Drugs	Yes	Director General appointed by the Ministry of Health	No.	Minimum (1)

^a^Indicates that based on the assessment, the NMRAs were considered to have a high degree of autonomy.

^b^The Pharmacoepidemiology Department within Caja contributes to the autonomy score for Costa Rica because its head is appointed by the Director of the CCSS, an institution separate from the Ministry of Health.

#### Evaluation of the degree of populism

Our study draws primarily on populism as operationalized by the Global Populism Database (GPD) project, which measures populism at the leader level. GPD is a speech-based approach and considers discourse as populist when it describes politics as a Manichean conflict between a virtuous people versus a corrupt elite ‘other’ (Hawkins et al. 2019). A sample of executive (president or prime minister) speeches is analysed and coded on a 0–2 scale based on the degree to which the speeches contain these elements, using a holistic method designed to capture latent elements of the text, including tone and style. It therefore generates a populism rhetoric score that combines expert coders’ evaluations of this phenomenon, with 0 being the least populist and 2 the most populist possible leader. Coders independently evaluate at least four speeches per leader in the original language. Scores are then averaged to produce a single populism score for each leader.

For each country, we selected the leader in power at the start of the COVID-19 pandemic and assigned the country the given point value from the GPD. Other populism measures, such as the Global Party Survey or the Varieties of Democracy project, code populism at the party level. Yet many of the leaders in our sample had limited legislative support, indicating that their parties were not highly relevant to policy outcomes. For instance, in Colombia and Guatemala, the respective presidents’ parties held <15% of seats in the legislature, and in Peru, the president dismissed Parliament in 2019 and governed without a party. To reflect this, we also report the percentage of seats that the president’s party held in the lower legislative chamber as of 2020. Finally, GPD also reports a measure of the left or right political ideology of the executive (scaled −1 to 1), which we also include in Table 3.

**Table 3:** Degrees of populist rhetoric of the political leader in the six countries studied.

Country	Political party^a^	President	Administration period	Populist rhetoric score^c^ (0–2)	Executive strength in legislature^d^	Left/right ideology^e^ (−1/1)
**Guatemala**	Vamos	Alejandro Giammattei	January 2020–January 2024	0.16	0.11	Right (1)
**Argentina**	Partido Justicialista/Frente de Todos	Alberto Fernández	December 2019–December 2023	0.29	0.47	Left (−1)
**Mexico**	MORENA	Andrés Manuel López Obrador	December 2018–October 2024	0.96	0.63	Left (−1)
**Colombia**	Partido Centro Democrático	Iván Duque	August 2018–August 2022	0	0.20	Right (1)
**Peru**	Not affiliated with a party^b^	Martín Vizcarra	March 2018–November 2020	0.14	0	Right (1)
**Costa Rica**	Partido Acción Ciudadana (PAC)	Carlos Alvarado Quesada	May 2018–May 2022	0	0.18	Left (−1)
**Region: median**				0.19		

^a^Political party of the president at the time of the pandemic (from December 2019 to August 2022).

^b^President Vizcarra originally served as Vice President of Pedro Pablo Kuczynski’s administration, affiliated with *Peruanos por el Kambio*, but post-2019 was not formally affiliated with any party.

^c^A scale from 0 to 2, where a higher score indicates stronger populist rhetoric.

^d^The percentage of seats held by the president’s party in the lower chamber of the legislature.

^e^The ideological values associated with the president, from the GPD data; options right, centrist, and left are coded from −1 (left) to 1 (right).

### Phase three: analysing causal mechanisms

The objective of Phase 3 was to assess the stakeholder map regarding COVID-19 kits in each country, and in doing so, provide a more nuanced account of causal mechanisms to complement our analysis of autonomy and populism, and to gain insights into other factors that may have influenced decisions to use COVID-19 kits containing ivermectin. We classify key institutions (e.g. medical associations) involved in decision-making during the pandemic and collect information on whether they were in favour of or against distributing COVID-19 kits (i.e. their position) and the extent of their influence on the decision (their power).

Using the information gathered in Phase 1 (local newspapers, press releases, official documents, social media, and peer-reviewed journals) that detailed the decisions about distributing COVID-19 kits, we developed an account of the decision-making process within each country and the specific role that each institution played in the pandemic response, including the NMRA and HTAO. We reviewed peer-reviewed literature and media reports describing which institutions were involved in the decision-making process. We also reviewed standard treatment guidelines to identify the institutions involved in authoring these documents. If the institution that we hypothesised to be involved was not mentioned in any documents on distributing COVID-19 kits, we consider it to have been absent from the decision-making process. We used this information to understand the role of the NMRA and HTAO in the decision to distribute COVID-19 kits.

### Phase four: cross-case comparison

We developed a matrix comparing the institutions involved, their degree of autonomy and power, and the outcome of decisions to distribute COVID-19 kits, to explore how different variables exerted heterogeneous influences across contexts and how each affected outcomes differently in each country.

We placed each institution according to its power level in the decision-making process. For example, in Guatemala, all medical associations are classified as low power, while in Colombia and Argentina, their power level is higher: in Guatemala, the associations did not influence decisions, while in Colombia, the Asociación Colombiana de Infectología (ACIN) was closely involved in the decision-making process and was even mentioned in public statements by President Ivan Duque.

For robustness, we conducted informational interviews with experts from each country working for the government either during the pandemic or currently. Experts were familiar with the decision-making process during the pandemic. They helped corroborate some of the findings from our document review, such as the extent to which we had accurately identified the decision-making process in each country and classified the power of each institution correctly.

## Results

We present results as follows. We first examine the NMRA’s degree of bureaucratic autonomy and the extent to which populist leaders in power explain each country’s decisions regarding the COVID-19 kit distribution. We then examine the decision-making process in each country in more detail.

### Bureaucratic autonomy and populism

**Figure 1:**
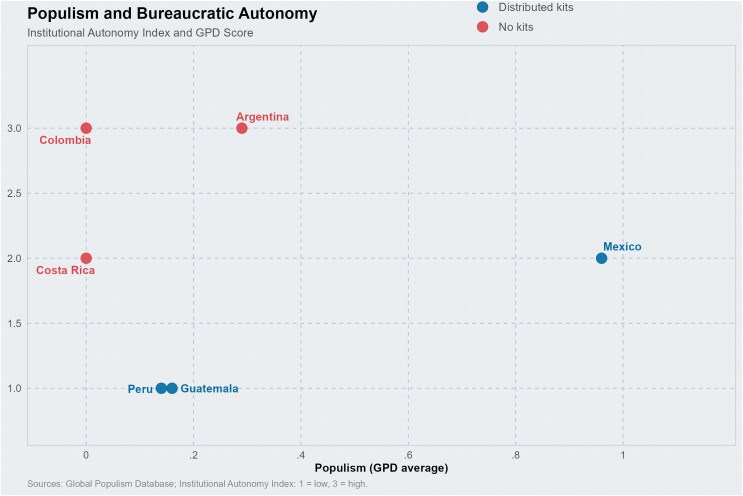
Populism and regulatory autonomy.

Figure 1 presents measures of bureaucratic autonomy and populism in relation to whether countries distributed COVID-19 kits. As shown, bureaucratic autonomy and populism partially explain the decisions of the six countries to distribute COVID-19 kits. The countries that distributed kits (Mexico, Peru, and Guatemala) are either low in autonomy (Peru, Guatemala) or medium in autonomy but high in populism (Mexico). The countries that did not distribute kits are either high in autonomy (Colombia and Argentina) or medium in autonomy but low in populism (Costa Rica).

Mexico is by a considerable margin the most populist country of the six, followed by Argentina. Guatemala and Peru are slightly below the regional median level of populism, while Colombia and Costa Rica have the minimum zero populism score. Argentina and Colombia have the highest levels of autonomy (3), suggesting institutions are positioned to guide evidence-based policy. Mexico and Costa Rica had intermediate levels (2), and Guatemala and Peru have the lowest values of bureaucratic autonomy for their NMRAs.

Colombia and Argentina did not distribute COVID-19 kits, which is congruent with the hypothesis that bureaucratic autonomy is an important determinant of evidence-based policymaking in this domain. For Colombia, the prediction from this theory is straightforward: President Ivan Duque was rated as minimally populist; together with Colombia’s autonomous NMRA, this suggests a low likelihood of distributing kits.

Argentina’s case is more complex: President Alberto Fernandez and the ruling Peronist *Frente de Todos* party had a relatively high level of populism (suggesting opposition to evidence-based policy); accordingly, Fernandez did personally support kit distribution, but Argentina did not move forward with this policy, suggesting the important role of autonomous scientific agencies.

Although both Costa Rica and Mexico had moderate NMRA autonomy scores, their decisions about distributing COVID-19 kits differed, suggesting the importance of populism. Costa Rica, with minimal populism, decided not to distribute COVID-19 kits. Mexico, with the highest populism index among the six countries studied, did distribute COVID-19 kits.

Guatemala and Peru scored low in NMRA autonomy and relatively low in populism. Despite limited populism, both Peru and Guatemala distributed COVID-19 kits. We consider this situation in more detail below.

### Interests, stakeholders, and the decision-making process in each country

To better understand the decision-making process in each case, we trace it by summarising the positions of the key stakeholders in each country. Table 4 presents a summary of key decision-makers in each country, including the President, Health Minister, Director of Social Security/Vice Minister, Medical Associations, Public Healthcare Providers, National Regulatory Agencies, and HTAOs.

**Table 4:** Overview of national and local stakeholders^a^.

Category	Guatemala	Costa Rica	Mexico	Colombia	Peru	Argentina
**President**	Alejandro Giammattei	Carlos Alvarado	Andrés Manuel López Obrador	Ivan Duque	Martín Vizcarra	Alberto Fernández
**Health Minister**	Amelia Flores Francisco Coma Martin	Daniel Salas	Oliva López Arellano^b^	Fernando Ruiz Gómez	Victor Zamora	Ginés González García
**Director of Social Security/Vice Minister**	Not specified	Roman Macaya	Zoé Robledo	Luis Alexander Moscoso	Fiorella Molinelli	Not specified
**Medical Associations**	GAID GIMA CMAG CPSG	Not specified	Mexican Associations of Infectious Diseases and Clinical Microbiology (MAIDCM) Mexican College of Critical Medicine (MCCM)	Colombian Association of Infectious Diseases (ACIN)	Medical College of Peru	Argentine Society of Infectious Diseases (SADI)
**Public Healthcare Provider**	Ministry of Public Health and Social Assistance (MPHSA)	Caja Costarricense de Seguro Social (CCSS)	Mexican Institute of Social Security (IMSS)	Ministry of Health and Social Protection (MHSP) Health Promoting Entities (EPS) Health Service Provider Institutions (IPSS)	Minsa/EsSalud	Ministerio de Salud Argentina
**National Regulatory Agency**	DRCPHP	DRPIS Pharmaco-epidemiology Directorate (Pharm)	Federal Commission for the Protection Against Sanitary Risks (COFEPRIS)	National Institute for Food and Drug Surveillance (Invima)	General Directorate of Medicines, Supplies, and Drugs (DIGEMID)	National Administration of Drugs, Food, and Medical Technology (ANMAT)
**Health Technology Assessment Organizations**	Not specified	National Center for Biotechnological Innovations	National Center for Technological Excellence in Health (CENETEC)	Institute for Technological Evaluation of Health (IETS)	CONCYTEC IETSI	National Commission for Evaluation of Health Technologies (CONETEC)

^a^GAID = Guatemala Association of Infectious Diseases; GIMA = Guatemalan Internal Medicine Association; CMAG = Critical Medicine Association of Guatemala; CPSG = College of Physicians and Surgeons of Guatemala; DRCPHP = Department of Regulation and Control of Pharmaceutical and Related Products; DRPIS = Department of Regulation of Products of Sanitary Interest; CONCYTEC = National Council of Science, Technology, and Technological Innovation; IETSI = Institute for Health Technology Assessment and Research.

^b^Local Health Minister (Mexico City).

The positions of key stakeholders and the subordination of NMRAs help us understand the outcomes in Guatemala and Peru, where kits were distributed despite non-populist governments. In Guatemala, the actors with the highest level of power, the President and Minister of Health, favoured using COVID-19 kits. Consequently, the Ministry of Public Health and Social Assistance (MPHSA) supported COVID-19 kits, as did Guatemala’s NMRA, the Department of Regulation and Control of Pharmaceutical Products, which is under the authority of the MPHSA. Major medical associations opposed the government distribution of COVID-19 kits, but the Ministry ignored their views.

A similar situation prevailed in Peru, where President Martin Vizcarra was responsible for distributing COVID-19 kits in early 2020. President Martín Vizcarra, Health Minister Victor Zamora, and Social Security Director/Vice Minister Fiorella Molinelli all favoured using COVID-19 kits. Aligning with the opinion of their directors, the Social Security institute in Peru (EsSalud), the Ministry of Health (Minsa), and, as part of it, Peru’s NMRA [the General Directorate of Medicines, Supplies and Drugs (DIGEMID)] were all in favour of using COVID-19 kits. As in Guatemala, medical associations vehemently opposed the use of COVID-19 kits, but the Ministry overruled them.

In Mexico, multiple states and institutions distributed COVID-19 kits, most notably Mexico City and the federal Mexican Social Security Institute (IMSS), the largest health care provider in the country. In Mexico City, the current Mexican President, Claudia Sheinbaum (then Mexico City’s mayor), was a staunch supporter of COVID-19 kits and endorsed their use. Oliva Lopez Arellano, Mexico City’s Health Minister, also favoured using COVID-19 kits, as did Zoe Robledo, Director of IMSS. Notably, technical agencies such as COFEPRIS, Mexico’s NMRA, and the National Center for Technological Excellence in Health, as well as the Mexican HTAO, were not involved in the decision to distribute COVID-19 kits. The constitutional authority responsible for emergencies, the General Health Council, did not issue any specific recommendations during the pandemic. Finally, the medical associations frequently criticised the government’s decision, but (as in Guatemala and Peru) decision-makers did not consider their views.

By contrast, in Costa Rica, all stakeholders opposed the use of COVID-19 kits. The President, the Health Minister, and the Director of the Social Security Provider agreed to follow scientific evidence and, together, decided to avoid using COVID-19 kits. The NMRA under the Ministry of Health in Costa Rica [Department of Regulation of Products of Sanitary Interest (DRPIS)], and health providers were also aligned with the decision-makers. The NMRA, DRPIS, is part of the Ministry of Health, while the Pharmacoepidemiology Department, the institution that guides health technology decisions regarding medicines within the ‘Caja’, has no financial independence (Tinoco-Mora 2005). Yet in Costa Rica’s universal healthcare system, under their sole healthcare services provider, ‘Caja,’ President Carlos Alvarado allowed technical and scientific leaders to lead the pandemic response, with consistent communication between Health Minister Daniel Salas and the Caja Director Roman Macaya (UN Sustainable Development Group 2020).

In Colombia, the President, the Health Minister, and the Vice Minister encouraged policymakers to heed the recommendations of the NMRA [National Institute for the Surveillance of Drugs and Food (Invima)] and the HTAO [Institute for Technological Evaluation of Health (IETS)], which opposed COVID-19 kits. Medical associations were also opposed to the use of COVID-19 kits. The unified advice against ivermectin from the Ministry of Health, IETS, Invima, and ACIN was taken seriously, and the federal government refrained from distributing COVID-19 kits (Redacción Semana 2020). When the municipal government of Cali decided to distribute COVID-19 kits, the federal government organized press conferences in which all technical agencies spoke out against the use of ivermectin without evidence and encouraged Cali to conduct clinical studies before disseminating COVID-19 kits (Redacción El Tiempo 2020, López-Medina et al. 2021). Moreover, IETS conducted ongoing appraisals of scientific evidence to provide up-to-date advice to decision-makers (Instituto de Evaluación Tecnológica en Salud 2020, Maritano 2020).

In Argentina, despite strong support from the President and the two Ministers of Health (both members of populist parties), COVID-19 kits were not distributed. The HTAO [National Commission for Evaluation of Health Technologies (CONETEC)] played a crucial role in influencing other agencies to recommend against the use of ivermectin. Furthermore, the NMRA, known as ANMAT, opposed its use, as did the medical associations. Two essential decision-makers were Minister of Health Carla Vizzoti and Council Advisor Sonia Gabriela Tarragona, who endorsed science and followed the recommendations of CONETEC. Both CONETEC and ANMAT institutions are substantially autonomous.

More detailed information on key decision-makers in each country is available in the Appendix (see online supplementary material).

## Discussion

### Institutional autonomy, interests, and populism as factors influencing the distribution of COVID-19 kits

Our results show a complex relationship between the autonomy of a country’s NMRA or HTAO, the degree of populism of its leader, the role of key decision-makers, and the distribution of COVID-19 kits. In relatively few cases, these theories make clear predictions, because countries rarely fall entirely into either the autonomous/pluralist quadrant (likely to follow evidence) or the non-autonomous/populist quadrant (likely to reject evidence). In our sample, the only such country was Colombia. Instead, the findings suggest a conditional relationship: bureaucratic autonomy appears to matter most when populist pressures are high, acting as an institutional buffer that can moderate political interference in evidence-based decision-making. However, bureaucratic autonomy is not always sufficient to prevent kit distribution: strong populism can marginalize technical agencies, as in Mexico. Nor is a populist government a necessary condition for governments to choose to distribute ineffective kits: both Peru and Guatemala distributed kits in the absence of populist leaders, but with strongly subordinated technical agencies.

### Autonomous institutions as a counterweight to populist pressure

The case of Argentina illustrates how autonomous regulatory institutions can moderate the influence of populist leadership on health policy decisions. Argentina had a populist ruling party and leader, yet did not distribute COVID-19 kits at a national level. The strength and autonomy of its technical institutions seemed to provide a countervailing force in the policy sphere, allowing this country to avoid distributing COVID-19 kits. Autonomous NMRAs and HTAOs provided a source of neutral, scientifically grounded advice that was structurally insulated from direct executive control. For example, in Argentina, CONETEC advised against using ivermectin (CONETEC 2021), and per these recommendations ivermectin was not adopted or distributed nationally despite a very populist government (in which the president himself had stated that medicines such as ivermectin would have a crucial positive effect). Interestingly, Argentina was the only country in our sample with public institutions in favour and against, with its NMRA and HTAO working to counter pressure from Argentina's president to distribute COVID-19 kits (López 2021). Thus, Argentina similarly illustrates how autonomous technical institutions can contribute to counterbalancing populist political pressures.

### Pluralist leadership and alignment with scientific evidence

Costa Rica and Colombia represent different pathways to the same outcome, in which moderate or strong institutional autonomy, combined with non-populist leadership, led to alignment with scientific recommendations. The Costa Rican case suggests that high levels of autonomy may not be necessary when political leaders are themselves oriented towards evidence-based governance and show a degree of pluralism. The Colombian case illustrates synergy between non-populist leadership and institutional autonomy. President Duque and his Partido Centro Democrático were not populist but could have chosen non-evidence-based policies. For example, the city of Cali chose to distribute kits. Yet, as noted above, Duque’s government took active measures to discourage this practice.

Taken together, Argentina, Colombia, and Costa Rica suggest multiple pathways to evidence-informed policy: either autonomous institutions that can resist populist pressure (Argentina), pluralist leaders willing to empower technical agencies despite limited formal autonomy (Costa Rica), or non-populist leaders well aligned with autonomous technical agencies (Colombia).

### Institutional vulnerability under populist governance

Peru, Mexico, and Guatemala had NMRAs that were under the Ministers of Health and lacked financial and operational independence, which may have contributed to their diminished ability to oppose decisions by political officials to promote COVID-19 kits. Populism played a role in Mexico, but not in Guatemala or Peru.

Mexico’s leader had the highest populism score among all six countries. Mexico’s national scientific institutions, such as COFEPRIS, have a medium level of autonomy, but this populist governing style may have contributed to the decision of Mexico’s leaders to sideline technical agencies (Sánchez-Talanquer et al. 2020). In fact, our stakeholder analysis showed that the government lowered the degree of autonomy of Mexico’s NMRA, COFEPRIS: it was autonomous from the Ministry of Health until 2020, when it was incorporated into the Ministry of Health organizational structure within the control of the Vice Minister of Health (López-Dóriga 2020). As noted previously, despite its technical mandate, COFEPRIS did not play a decisive role in deciding whether to distribute COVID-19 kits.

### Populist policies without populists?

Neither Peru nor Guatemala had a populist government, but both governments distributed ivermectin. Both cases illustrate the role of interest groups. In Peru, the NMRA’s low level of autonomy paved the way for using ivermectin, once President Vizcarra chose this course (El Peruano 2020). This was not without controversy: multiple physicians and health experts resigned due to constant conflicts with Minister of Health Victor Zamora, who ignored the groups of experts he already had at his disposal (Castañeda Palomino 2020). Furthermore, a conflict between EsSalud and one of Peru’s HTAOs, Institute for Health Technology Assessment and Research (IETSI), which advised against using ivermectin, ended with the firing of two scientists (Torres 2020). Medical associations demanded that the government retract its decision in favour of ivermectin while EsSalud was giving COVID-19 kits to their population. However, the NMRA, DIGEMID, followed the President and Health Minister's demands.

In Guatemala, the regulatory agency is not autonomous. Technical institutions were deliberately undermined by political leaders; e.g. a group of experts called COPRECOVID, created to take on multiple responsibilities at the beginning of the pandemic, was dissolved in 2020, and its duties were transferred to the Ministry of Health (Larios 2021). As noted above, when President Giammattei and his Minister of Health decided to distribute COVID-19 kits, technical agencies had little scope to oppose these decisions.

There is a notable similarity to the Peru and Guatemala cases, which may help explain their presidents’ choice of COVID-19 kits despite not being populist. While divided government is common in Latin America’s presidential systems (Mainwaring 1993), in Peru and Guatemala, the president had very small to non-existent bases in Congress (see Table 1). Poorly institutionalized, personalist governance may be an alternative way for short-run, non-evidence-based solutions like COVID-19 kits to enter the picture. Just as autonomous bureaucracies constrain executive policymaking by pushing executives to consider evidence, political scientists focused on Latin America have argued that institutionalized parties also constrain executive policymaking because the executive is embedded in a larger institution with expertise, connections to voters, and institutionalized rules (Mainwaring and Scully 1995, Spiller et al. 2008). Presidents like Vizcarra and Giammattei lacked the benefits of institutionalized parties and had to find ways to communicate directly with voters. Even in the absence of populist ideology, this may open up the field for short-term fixes like COVID-19 kits.

While our comparative case study research design does not allow us to draw causal conclusions, our study highlights the complex processes that lead to policy choices in this set of cases. We observe a contingent relationship between populist politics and the probability that governments choose non-evidence-based policies, such as the distribution of COVID-19 kits. Our design allows us to observe the mechanisms at play, including the role of bureaucratic autonomy, which highlights the important role of the autonomy of NMRAs in working as checks and balances to the potential wave of populism and interest groups.

However, we also see the critical role of individual decision-makers. In some cases, technically competent officials may align with populist leaders, possibly in order to advance within party hierarchies, while those who resist may be sidelined or removed, as was illustrated by the dismissal of IETSI researchers in Peru. In other cases, politicians may show surprising commitment to evidence-based policies. This suggests that the influence of individual decision-makers is itself mediated by both personal characteristics and political incentives, which our study captures only partially.

### Policy implications

Although this study focuses on the distribution of COVID-19 kits, the cases suggest policy implications for broader governance during health crises. First, autonomous regulatory and HTAO institutions may help preserve evidence-based decisions when political pressures from interest groups favour quick interventions. In cases where such institutions retained influence, governments were more likely to align with scientific guidance. Second, institutional autonomy appears most effective when technical agencies are formally integrated into crisis decision-making structures. In cases where COVID-19 kits were distributed, technical bodies were sidelined or subordinated to political authorities. Third, the findings highlight the political appeal of low-cost, easily distributable technologies during crises. Strengthening rapid evidence appraisal processes, paired with autonomous technical bodies’ participation in the decision-making process, may reduce the risk of adopting ineffective interventions in the face of uncertainty.

However, these implications should be interpreted cautiously. Autonomy alone does not guarantee evidence-based policymaking.

### Broader implications of our findings

A document published by the WHO, ‘Preparedness and Resilience for Emerging Threats (PRET)’, does not mention the need to strengthen decision-making in national agencies such as regulatory agencies and agencies that evaluate new technologies (World Health Organization 2023b). Our findings suggest the importance of autonomous regulatory agencies in ensuring effective decision-making and evaluating the impact of emerging healthcare technologies. It would also be valuable to appraise the political context of countries to understand the policy space of technical institutions better. Currently, there is little systematic analysis of the political context to inform pandemic responses, including the positioning of technical institutions and strategies for enhancing their autonomy and integration into crisis decision-making.

Relying on ivermectin as a treatment for COVID-19 was a waste of resources that potentially endangered the population without benefits. Regardless of the number of cases and deaths, countries in Latin America used COVID-19 kits despite the WHO stating that ivermectin was ineffective. Using COVID-19 kits as a last-resort alternative was not inevitable; policymakers had other, more effective, evidence-based alternatives.

## Limitations

This study has limitations, notably that the sample of six countries limits our ability to draw conclusions applicable to the rest of the region or provide a causal interpretation of the suggestive patterns we have identified regarding the relationship between bureaucratic autonomy, populist politics, and evidence-based decision-making. Furthermore, despite gathering information from each country, understanding the actual decision-making process through a literature review can be challenging, even when supplemented by confirmatory information gathered from interviews. This could affect our results. Also, the lack of data availability could hinder a more comprehensive understanding of the decision-making process.

A further limitation of our populism measure is its reliance on the GPD. The GPD’s holistic speech-coding method relies on subjective coder judgment. In addition, by focusing on discourse, the GPD measures rhetoric rather than behaviour in office. Leaders in our sample could score high on populism with regard to their speech content, but govern quite differently, or vice versa.

Several alternative explanations may also have influenced the decision to distribute COVID-19 kits. Countries differed in epidemiological burden, fiscal space, health system responsiveness, and access to technical expertise, which may have shaped the appeal of low-cost or rapidly deployable interventions, even when potentially harmful. However, the WHO issued guidelines recommending other low-cost, rapidly deployable, evidence-based interventions. The appeal of COVID-19 kits may therefore reflect not only resource constraints but also the tendency of populist leaders to promote simple, visible solutions that resonate with anti-elite narratives, such as the notion that effective cures have been suppressed by powerful interests (Lasco 2020, Lasco and Gregory Yu 2022).

## Conclusion

This study examined decision-making related to the distribution of COVID-19 kits in six Latin American countries despite international scientific guidance advising against their use. By comparing six countries exposed to similar regional conditions but differing in political and institutional characteristics, we explored how bureaucratic autonomy, populism, and stakeholder interests influenced the decision whether to distribute COVID-19 kits.

Our findings suggest that neither institutional variables, such as the level of bureaucratic autonomy, nor ideological factors, such as the degree of populism, nor stakeholder interests alone determined the decision about whether or not to distribute COVID-19 kits. Countries combining populist leadership with weakly autonomous regulatory institutions were more likely to distribute COVID-19 kits, whereas autonomous regulatory and health technology assessment institutions were sometimes able to moderate political pressures and support evidence-informed decisions. Countries with autonomous NMRAs were better positioned to resist political pressure, even under populist leadership, while countries lacking such institutional buffers were more susceptible to adopting interventions unsupported by scientific evidence, even when led by non-populist leaders and parties.

Appraising the political context of each country to better understand the policy space for technical institutions could inform pandemic responses. Strengthening the autonomy of regulatory and assessment agencies may enhance governments’ ability to evaluate emerging health technologies during future emergencies. Efforts to strengthen pandemic response will require action across multiple domains, including steps to protect institutional autonomy and to build broad-based coalitions for evidence-based policy.

## Supplementary Material

czag069_Supplementary_Data

## Data Availability

The data underlying this article are available in the article and in its online supplementary material.

## Reflexivity statement

Both J.A.R.D. and V.J.W. have extensive professional and academic experience in Latin America. J.A.R.D. was born, raised, and medically trained in Mexico, where part of the research was conducted; he is also doing a PhD focusing on the Mexican Healthcare System. V.J.W. lived in Mexico for several years and worked on pharmaceutical policy and regulation. This satisfies *Health Policy and Planning*’s authorship policy regarding low- and middle-income countries-based research. The author team includes researchers based in Latin America, the United States, and the United Kingdom. This composition ensures both regional familiarity with health system governance in Latin America and methodological rigour in health policy analysis. The authors represent different genders and levels of seniority, combining academic, clinical, and policy expertise. Collaboration aimed to maintain inclusivity and balance in perspectives during data collection, analysis, and interpretation.
