# Supplementary material for: COVID-19 kit distribution in six Latin American countries: bureaucratic autonomy, populism, and evidence-based policymaking
Source: Health Policy Plan. 2026 May 20;41(7):1165–78. doi: 10.1093/heapol/czag069 (PMC13481690; doi:10.1093/heapol/czag069)
Supplement: Supplementary file 1 [file czag069_supplementary_data.zip › Annex.docx]

**Annexe.**

The results are divided according to country.

*Guatemala*

*Decision-makers:* In Guatemala, we identified that the President, Alejandro Giammattei, and Minister of Health Amelia Flores (who resigned on September 16, 2021) (Mendoza, 2021) were in favor of using COVID-19 kits, including ivermectin. (Gobierno de Guatemala, 2020; Morales, 2021; Román et al., 2020)

*Public Health Provider:* The institution in charge of distributing the COVID-19 kits is the Ministry of Public Health and Social Assistance, specifically the Drug Accessibility Program (PROAM). The distribution of kits started in August 2020 (Salud con lupa, 2020), and by 2022, Eduardo Vargas, Director of PROAM, stated that the program had distributed more than 500,000 kits. (Larios, 2022)

*National Regulatory Authority:* The Department of Regulation and Control of Pharmaceutical and Related Products, a branch of the General Directorate of Health Regulation, Surveillance, and Control of the Ministry of Public Health and Social Assistance, both signed and approved the use of COVID-19 kits, including ivermectin.(Ministerio de Salud Pública y Asistencia Social, n.d.)

*Health Technology Assessment Organizations:* We did not identify any Health Technology Assessment Organization in Guatemala nor inside the Ministry of Public Health and Social Assistance.

*Medical Associations:* The College of Physicians and Surgeons of Guatemala, the Guatemalan Internal Medicine Association, and the Critical Medicine Association of Guatemala submitted a statement on July 14, 2020, which showed their discontent with the position that the Ministry had taken to distribute ivermectin to the general population. (Ola, 2020) In addition, the Guatemalan Association of Infectious Diseases submitted a separate statement the same day, advising against the use of ivermectin. (Ola, 2020)

*Costa Rica*

*Decision-makers:* The actors surrounding the decision to use COVID-19 kits in Costa Rica that we identified were President Carlos Alvarado, the Director of the Costa Rican Social Security (known as "*Caja*") Roman Macaya, and the Health Minister, Daniel Salas. (UN Sustainable Development Group, 2020) According to the United Nations, President Alvarado let Daniel Salas, alongside Roman Macaya, lead the response to fight COVID-19. (UN Sustainable Development Group, 2020)

*Public Health Provider***:** The "*Caja*" took a central role in the response to the pandemic. As their healthcare provider, they are responsible for treating citizens, but they pressured the government to adopt COVID-19 measures to protect the population. (The Tico Times, 2021) Since the early days of the pandemic, the medicines that were used at *"Caja"* were revised by a team of Pharmacoepidemiology experts known as the Pharmacoepidemiology Directorate (who work as a regulatory institution) within "*Caja*." (Tinoco-Mora, 2005)The Directorate analyzes publications from international organizations such as the Food and Drug Administration (FDA), the National Institute for Health and Care Excellence (NICE), and the European Medicines Agency (EMA). (Rodríguez and Ávalos Rodríquez, 2022) In addition, Health Minister Salas mentioned in an interview that they constantly reevaluated their decisions as new information was available.(Rodríguez and Ávalos Rodríquez, 2022) This explains why they started using hydroxychloroquine and convalescent plasma during the early months of the pandemic but quickly shifted their decision as further information was coming out. (Ávalos Rodríquez, 2020; Le Lous, 2021; Rodríguez, 2022) On the other hand, there is no report saying that Costa Rica distributed COVID-19 kits. Moreover, "*Caja*" has a program called Basic Comprehensive Health Care Teams (EBAIS), which follows the patients with in-house visits after being diagnosed with COVID-19 and provides the required medicines to patients in their own homes. (Brooks, 2020) Since *"Caja"* decided not to use COVID-19 kits, the EBAIS did not distribute them among those they visited at home.

*National Regulatory Authority:* Costa Rica has a Medical Regulatory Authority called the

Department of Regulation of Products of Sanitary Interest (DRPIS). (Ministerio de Salud, 2022) Nevertheless, we have not identified any information that the regulatory authority endorsed the use of COVID-19 kits. Their role relies on approving the distribution of medicines. The decision-making process based on scientific evidence involved internal sectors of "*Caja,*" more specifically, their Pharmacoepidemiology Directorate. (Rodríguez and Ávalos Rodríquez, 2022)

*Health Technology Assessment Organizations:* We did not find evidence that The National Center for Biotechnological Innovations (CENIBiot) and the National Technology Center (CENAT) were involved in a decision related to the use of COVID-19 kits.

*Medical Associations:* We did not identify any Health Medical Association involved in the decision to use COVID-19 kits in Costa Rica.

*Mexico*

*National decision-makers:* in Mexico, local decision-makers disagreed with the federal government on the use of COVID-19 kits. The state of Chiapas distributed 10,000 COVID-19 kits. (Luna, 2020) Also in Mexico City, home to over eight million Mexicans, the Mayor and decision-maker Claudia Sheinbaum, alongside her team, which included the local Minister of Health Oliva López Arellano, distributed COVID kits. (Camhaji, 2022)

Although not depicted in the stakeholder map, at the federal level, institutions issued position statements regarding COVID-19 kits after decisions had been made at a local level. (Hernández, 2021) The Ministry of Health^[1]*^ had a position towards ivermectin in August 2021 when they stated that ivermectin was not useful enough. (Redacción El Universal, 2022a) Nevertheless, the Social Security Institution (IMSS) defended using ivermectin. The Director of IMSS, Zoe Robledo, supported ivermectin throughout the pandemic. (Latinus, 2021a) Well-equipped organizations were sidelined and had no decision in the time of crisis. As an example, the General Health Council, considered the highest collegiate responsible for decision-making during health emergencies by the Constitution of Mexico, had a secondary role.(Consejo de Salubridad General, 2022; Sánchez-Talanquer et al., 2020) The council had its first meeting on March 19, 2020, 20 days after Mexico reported its first case. (Sánchez-Talanquer et al., 2020) Furthermore, last year, the Secretary of the council resigned due to differences with the Health Secretary.(Redacción El Universal, 2022b)

*Public Health Provider:* In December 2020, Mexico City started distributing COVID-19 kits that included ivermectin. (Sarabia, 2022) In 2022, it was revealed that alongside Mexico City's decision to give ivermectin, an agency that is not constantly involved in health decisions in Mexico, the Public Innovation Digital Agency (ADIP), members of the Mexican Institute of Social Security (IMSS), as well as Mexico City Health Minister, conducted a controversial study with serious ethical concerns that alerted the public and was eventually retracted. (Dyer, 2022) In consequence, President Lopez Obrador, Dr Jorge Alcocer, and Dr. Lopez Gatell came out to defend the steps taken by the government of Mexico City (Expansión Política, 2022; Redacción El Universal, 2022a) even though the Ministry of Health announced in August 2021 that they did not recommend using ivermectin.(Redacción El Universal, 2022a) There were even reports that said that Dr. Jorge Alcocer was against using ivermectin, but his lack of leadership did not prevent others from using ivermectin.(Ramírez Coronel, 2022) Despite the backlash, Mayor Sheinbaum, as well as her team, defended the results and continued using ivermectin for several months.(Krauze, 2022; Santiago, 2021)

*National Regulatory Authority:* During the COVID-19 pandemic, organizations that had been involved in other crisis periods, such as the National Institutes of Health or the Federal Commission for the Protection Against Sanitary Risks (COFEPRIS), did not take a leading role. (Sánchez-Talanquer et al., 2020) Their silence was notable, and recently, COFEPRIS rejected publishing the therapeutical information about ivermectin after a citizen asked about the matter, and federal authorities required COFEPRIS to disclose their evidence.(Redacción Animal Político, 2022)

*Health Technology Assessment Organization:* In Mexico, the National Center for Technological Excellence in Health (CENETEC) provides information based on the best available evidence for adequate decision-making in health technologies for health services in Mexico. (CENETEC, n.d.) CENETEC was affected by the austerity policies implemented by the administration of Lopez Obrador, diminishing its role during the pandemic. (Sánchez-Talanquer et al., 2020)

*Medical Associations:* After the scandal of the COVID-19 kits that included ivermectin in Mexico City, the Mexican Associations of Infectious Diseases and Clinical Microbiology and the Mexican College of Critical Medicine recommended against using ivermectin. The President of the Mexican College of Critical Medicine went so far as to say that the clinical evidence that the government was using did not have statistical significance that justified the use of ivermectin to treat COVID-19.(Hernández Mares, 2021)

*Colombia*

*National and subnational decision-makers***:** The Colombian government did not recommend the use of ivermectin as a treatment for COVID-19, according to a statement by the Ministry of Health.(Ministerio de Salud y Protección Social, 2020) In a statement released by The Ministry of Health and Social Protection, the government announced that they do not recommend the use of ivermectin as a treatment for COVID-19, except when used within a clinical study that has been approved by the National Institute for Food and Drug Surveillance (Invima). This statement was released by Vice Minister Luis Alexander Moscoso Osorio in the early months of the pandemic and followed the clinical guidelines released by PAHO at the time of the press release.(Ministerio de Salud y Protección Social, 2020; Organización Panamericana de la Salud, 2020) Moreover, the President at the time, Ivan Duque, released a statement on July 13, 2020, accompanied by the Minister of Health, Fernando Ruiz, in which they did not recommend the use of ivermectin outside clinical trials.(Redacción Semana, 2020) It is noteworthy to state the leadership shown by Fernando Ruiz and Marta Lucía Ospina (General Director of Colombia's National Health Institute) throughout the pandemic; they earned the trust of multiple organizations and Colombian citizens. Fernando Ruiz created a group of experts to understand the behavior of the pandemic. (Ministerio de Salud, 2020a) Although Cali, one of the most important cities in Colombia, decided to use ivermectin for their population, President Ivan Duque, the Ministry of Health, and Invima restated that they did not support using ivermectin outside clinical trials.(Redacción Semana, 2020) Eventually, researchers in Cali conducted a research study with some support from the local authorities and was approved by Invima. They concluded that there was no benefit to using ivermectin, and the results were published in JAMA. (López-Medina et al., 2021; Redacción El País, 2020)

*Public Health Providers:* We did not find any evidence that either the Health Promoting Entities (EPS) or the Health Service Provider Institutions (IPSS) released any statement regarding the use of COVID-19 kits in the public sector. Nevertheless, experts from the region stated that both were not supportive of using ivermectin.

*National Regulatory Authority:* The National Regulatory Agency in Colombia is Invima, which works as an adviser to the government and not as a regulatory agency per se. In conjunction with The Ministry of Health and Social Protection, Invima endorsed and retweeted the official statement of the Ministry of Health. Furthermore, they mentioned how ivermectin was withdrawn from a clinical study conducted by WHO (called Solidarity) because of the lack of safety and efficacy in treating patients with COVID-19.(Invima, 2020; Maritano, 2020) Since December 17, 2021, Invima has published on their website an article explaining why it is not recommended to use ivermectin for COVID-19. (Invima, n.d.)

*Health Technology Assessment Organizations:* To support the Ministry of Health and Social Protection in addressing the issue of ivermectin, the Institute for Technological Evaluation of Health (IETS) analyzed the position of 12 different agencies and international institutions respecting the use of ivermectin. According to the government press release, the IETS studied the WHO, Centers for Disease Control and Prevention (CDC), and the European Centre for Disease Prevention and Control (ECDC) to decide.(Instituto de Evaluación Tecnológica en Salud, 2020; Maritano, 2020; Ministerio de Salud y Protección Social, 2020)

*Medical associations:* The Colombian Association of Infectious Diseases (ACIN) was also part of this conjoint statement.(Ministerio de Salud y Protección Social, 2020) The ACIN released a separate statement on May 24, 2020, insisting that ivermectin should be used only for clinical trials.(Asociación Colombiana de Infectología, 2020)

*Peru*

*National Decision-makers:* The response of Peru to the pandemic varied over time. The political turmoil that the country has endured during recent years caused multiple Presidents and Health Ministers to be in charge of the pandemic. Since the start of the pandemic, until the moment of this research, Martín Vizcarra, Manuel Merino, Francisco Sagasti, Pedro Castillo, and Dina Boluarte have been Constitutional Presidents of the Republic of Peru. (The Associated Press, 2022) In addition, 11 different individuals have been Health Ministers in the past three years.(Redacción Vigilante, 2023) During the time of President Martín Vizcarra, Victor Zamora (a Health Minister) emitted a technical document alongside the National Institute of Health through the Public Health Evidence Generation Unit stating, "based on the discussion of the document, even when the level of evidence is low, and the majority of the members of the group in favor, it is put to the consideration of the treating physicians." (El Peruano, 2020) Moreover, Peru created different Health Expert Committees to advise the Health Minister. Nevertheless, multiple conflicts within the Committees emerged, causing several physicians to abandon the group. The reason tended to be discrepancies with Victor Zamora.(Castañeda Palomino, 2020) Furthermore, in June 2020, President Vizcarra announced 490,000 doses of ivermectin for individuals who tested positive for COVID-19, following the recommendation of Minsa (Minister of Health).(Redacción RPP, 2020a; Trigo et al., 2020) It was not until January 2021 when Pilar Mazzetti (the successor of Victor Zamora), under the presidency of Francisco Sagasti (who opposed using ivermectin) (Moreno, 2021), that Minsa recognized not to use ivermectin for the general population because it lacked the approval of international organizations. Although, physicians could use ivermectin according to their own expertise. (Redacción Perú21, 2021a) Finally, in March 2021, Oscar Ugarte (successor of Pilar Mazzetti) stated that Minsa would not use ivermectin and would stick to international guidelines. (Collave García, 2021; Redacción Perú21, 2021b)

*Public Health Providers:* The leading Public Health Provider in Peru is the Ministry of Health (Minsa), following the leadership of the Health Minister. (Elizabeth Alcalde-Rabanal et al., 2011) Likewise, the Public Sector includes a Social Security Institution called EsSalud. (Elizabeth Alcalde-Rabanal et al., 2011) Not only have we identified the use of ivermectin within Minsa institutions but also in EsSalud. (Agencia Peruana de Noticias Andina, 2020; Redacción El Comercio, 2020) Moreover, the general Director of EsSalud, Fiorella Molinelli, took a leading role, clashing with other institutions. (Torres, 2020)

*National Regulatory Authority:* The General Directorate of Medicines, Supplies, and Drugs (DIGEMID) is the national authority within the Ministry of Health, dependent on the Vice Ministry of Public Health, in charge of pharmaceuticals, medical devices, and health products. (Ministerio de Salud, 2023) As it is an institution within Minsa, this paper has not identified any opposition from part of DIGEMID to use COVID-19 kits. We identified that in June 2020, DIGEMID published an announcement to gather professional experts capable of manufacturing ivermectin. (Ministerio de Salud, 2020b)

*Health Technology Assessment Organizations*: The National Council of Science, Technology, and Technological Innovation (CONCYTEC) is the governing body in charge of directing, promoting, coordinating, supervising, and evaluating State actions throughout the country in the field of science, technology, and technological innovation.(Consejo Nacional de Ciencia, n.d.) This research did not identify any statement from CONCYTEC endorsing the use of ivermectin. On the other hand, the Institute for Health Technology Assessment and Research (IETSI) is the institution in charge of the "generation of scientific, economic and social evidence" under the leadership of EsSalud. (IETSI, n.d.) Their vision is to inform decision-making in health and optimize the use of resources in order to achieve and extend quality health services for Peruvians through Social Security. (IETSI, n.d.) Despite their role (limited to only Social Security), on May 8, 2020, after the Minister of Health published the technical document in favor of ivermectin, IETSI published a report maintaining their position not to use ivermectin to treat COVID-19 and to use it only in clinical trials. (IETSI, 2020) In October 2020, IETSI published an article stating that using ivermectin, hydroxychloroquine, and azithromycin, all medicines included in the COVID-19 kits, increased mortality by 84%.( Soto-Becerra et al., 2020) As a consequence, Fiorella Molinelli, Director of EsSalud, fired Patricia Pimentel, IETSI general director, as well as Yamilee Hurtado, research director of IETSI and principal researcher of the research paper. (Torres, 2020) The decision was backed by the President at the moment, Martín Vizcarra. (Redacción RPP, 2020b)

In the end, in August 2021, EsSalud followed IETSI advice and changed its clinical guidelines, disadvising the use of ivermectin months after Minsa had discontinued its use in their own hospitals. (Collave García, 2021; IETSI, 2021)

*Medical associations:* We have identified two instances in which medical associations have expressed their thoughts or recommended Minsa to avoid using ivermectin to treat COVID-19. The first case was in June 2020, when a group of 12 physicians wrote a letter to Minsa advising them not to use ivermectin. (La República, 2021) Furthermore, in January 2021, the Medical College of Peru, through their dean, Dr. Palacios Celi, stated that they disapproved of using ivermectin. (Redacción RPP, 2021)

*Argentina*

*Decision-makers:* The most important decision-makers in Argentina were President Alberto Fernandez, as well as Health Minister Ginés González. The President stated that such medicines were going to have a crucial effect. (Bermejo, 2020) Nevertheless, despite his comments, it is interesting that we have not identified COVID-19 kits that were given to the population. In other words, the Federal government did not distribute ivermectin as a preventive measure. Two crucial decision makers were Carla Vizzoti (as Health Minister) and Sonia Gabriel Tarragona. Both endorsed science and followed the recommendations of the National Commission to Evaluate Health (CONETEC). In contrast with the federal government, at least five local governments used ivermectin in hospitals despite not having the approval of the National Regulatory Authority called the National Food, Drug, and Medical Technology Administration (ANMAT), nor following the recommendations of CONETEC. (López, 2021) Some regions, such as Tucuman, went so far as to conduct their own research study, led by the local Health Minister, and presented dubious results (a significant reduction of symptoms after using ivermectin). (Chahla et al., 2022)

*Public Health Providers:* This article did not find any evidence that the Public Health Providers released any statement regarding the use of ivermectin in the public sector.

*National Regulatory Authority:* The National Food, Drug, and Medical Technology Administration (ANMAT) has never approved ivermectin. Their stance has remained fixated throughout the pandemic, stating that there is a lack of compelling evidence to favor the use of ivermectin. (Ámbito, 2021) Despite their position being in line with other National Regulatory Authorities, multiple citizens in various regions presented appeals to the court to make ANMAT declare ivermectin as a valid treatment for COVID-19. (Canonico, 2021; Rosario3, 2021)

*Health Technology Assessment Organizations:* The National Commission to Evaluate Health Technologies (CONETEC) is the health authority on the incorporation, form of use, financing, and coverage policies of health technologies. (CONETEC, 2023) CONETEC conducted evidence-based guidelines assessing the effectiveness of several medicines, among them ivermectin. According to the evidence that we have gathered, CONETEC did not approve ivermectin at any point, becoming the staunchest critic of using the drug, as well as advising other institutions to avoid using ivermectin. (CONETEC, 2021)

*Medical associations*: The Argentine Society of Infectious Diseases (SADI) stated that the available evidence on whether to use ivermectin is of "very low quality so far and does not have reported clear clinical efficacy data" while mentioning that drugs come with potential risks and ivermectin "is not a harmless drug." Despite the position taken by the government, SADI recommended that "as long as there is no concrete evidence of higher quality, duly registered and ethically acceptable, it should not be used for the treatment or prophylaxis of SARS-CoV-2. ʺ(Vet Market, 2021

**References:**

Agencia Peruana de Noticias Andina, 2020. EsSalud y FFAA preparan 100,000 kits para atender la pandemia en regiones [WWW Document]. Andina. URL https://andina.pe/agencia/noticia-coronavirus-essalud-y-ffaa-preparan-100000-kits-para-atender-pandemia-regiones-807594.aspx (accessed 8.8.22).

Ámbito, 2021. ¿Qué es la ivermectina y por qué la ANMAT no aprueba su uso contra la Covid-19? [WWW Document]. Ámbito. URL https://www.ambito.com/informacion-general/covid-19/que-es-la-ivermectina-y-que-la-anmat-no-aprueba-su-uso-contra-la-n5161630 (accessed 4.17.23).

Asociación Colombiana de Infectología, 2020. RECOMENDACIONES PARA EL MANEJO DE INFECCIÓN POR SARS CoV-2-COVID-19.

Ávalos Rodríquez, Á., 2020. Falta de protocolo obliga a CCSS a suspender hidroxicloroquina para enfermos de covid-19 [WWW Document]. La Nación. URL https://www.nacion.com/el-pais/salud/ccss-suspende-de-manera-temporal-la/ZFFULKHY6ZBCDI2GKXI4SHVRM4/story/ (accessed 3.15.23).

Bermejo, L., 2020. Los dos remedios argentinos para tratar el coronavirus de los que habló Alberto Fernández [WWW Document]. Página12. URL https://www.pagina12.com.ar/297220-los-dos-remedios-argentinos-para-tratar-el-coronavirus-de-lo (accessed 4.12.23).

Brooks, D., 2020. Coronavirus en Costa Rica: cuál es la efectiva fórmula en el país de América Latina donde mueren menos pacientes de covid-19 [WWW Document]. BBC News Mundo. URL https://www.bbc.com/mundo/noticias-america-latina-52480615 (accessed 3.15.23).

Camhaji, E., 2022. El reparto de ivermectina vuelve a sacudir a México | EL PAÍS México [WWW Document]. El País México. URL https://elpais.com/mexico/2022-02-08/el-reparto-de-ivermectina-vuelve-a-sacudir-a-mexico.html (accessed 2.21.23).

Canonico, P., 2021. Dos científicos argentinos que investigaron la ivermectina defienden su uso contra el coronavirus [WWW Document]. Clarín. URL https://www.clarin.com/sociedad/cientificos-argentinos-investigaron-ivermectina-defienden-uso-coronavirus_0_sAmPqQZGN.html (accessed 4.12.23).

Castañeda Palomino, G., 2020. Seis médicos han renunciado al comité de expertos del COVID-19 que apoya al Minsa [WWW Document]. El Comercio. URL https://elcomercio.pe/lima/sucesos/seis-medicos-han-renunciado-al-comite-de-expertos-del-covid-19-que-apoya-al-minsa-noticia/ (accessed 10.31.23).

CENETEC, n.d. Centro Nacional de Excelencia Tecnológica en Salud [WWW Document]. gob.mx. URL https://www.gob.mx/salud/cenetec (accessed 4.6.23).

Chahla, R.E., Ruiz, L.M., Mena, T., Brepe, Y., Terranova, P., Ortega, E.S., Barrenechea, G.G., Goroso, D.G., Bruno, M. de los Á.P. de, 2022. Ivermectin repurposing for COVID-19 treatment of outpatients with mild disease in primary health care centers. Research, Society and Development 11, e35511830844–e35511830844. https://doi.org/10.33448/RSD-V11I8.30844

Collave García, Y., 2021. Minsa ya no usará la ivermectina para tratar el COVID-19: ¿qué dice la ciencia sobre su eficacia para los pacientes? [WWW Document]. El Comercio. URL https://elcomercio.pe/tecnologia/ciencias/minsa-ya-no-usara-la-ivermectina-para-tratar-el-covid-19-que-dice-la-ciencia-sobre-su-eficacia-para-los-pacientes-noticia/ (accessed 4.4.23).

CONETEC, 2023. CONETEC | Argentina.gob.ar.

CONETEC, 2021. Ivermectina para el tratamiento de pacientes con COVID-19 y expuestos al SARS-CoV-2 ACTUALIZACIONES BASADAS EN EVIDENCIA Ivermectina para el tratamiento de pacientes con COVID-19 y expuestos al SARS-CoV-2.

Consejo de Salubridad General, 2022. Secretario | Consejo de Salubridad General [WWW Document]. URL http://www.csg.gob.mx/consejo/secretario.html (accessed 3.22.23).

Consejo Nacional de Ciencia, T. e I.T., n.d. Información institucional - Consejo Nacional de Ciencia, Tecnología e Innovación Tecnológica - Plataforma del Estado Peruano [WWW Document]. gob.pe. URL https://www.gob.pe/institucion/concytec/institucional (accessed 10.31.23).

Dyer, O., 2022. Covid-19: Mexico City gave ivermectin kits to people with covid in "unethical" experiment. BMJ 376, o453. https://doi.org/10.1136/BMJ.O453

El Peruano, 2020. Modifican el Documento Técnico: Prevención, Diagnóstico y Tratamiento de personas afectadas por COVID-19 en el Perú - RESOLUCION MINISTERIAL - N° 270-2020-MINSA - PODER EJECUTIVO - SALUD [WWW Document]. El Peruano. URL https://busquedas.elperuano.pe/normaslegales/modifican-el-documento-tecnico-prevencion-diagnostico-y-tr-resolucion-ministerial-n-270-2020-minsa-1866159-4/ (accessed 8.8.22).

Elizabeth Alcalde-Rabanal, J., Lazo-González, O., Nigenda, G., 2011. Sistema de salud de Perú. Salud Publica Mex 53, 243–254.

Expansión Política, 2022. Críticas por ivermectina son parte de campaña contra el gobierno, dice AMLO [WWW Document]. Expansión Política. URL https://politica.expansion.mx/mexico/2022/02/08/criticas-al-uso-de-ivermectina-son-ataques-al-gobierno-amlo (accessed 2.21.23).

Fonseca, D., 2020. El factor López-Gatell - The New York Times [WWW Document]. The New York Times. URL https://www.nytimes.com/es/2020/07/23/espanol/opinion/lopez-gatell.html (accessed 4.6.23).

Gobierno de Guatemala, 2020. En total, se elaborarán 1 millón de Kits COVID-19 que serán entregados a pacientes leves con coronavirus en todo el país. #JuntosSaldremosAdelante... | By Gobierno de Guatemala [WWW Document]. Facebook. URL https://www.facebook.com/guatemalagob/videos/en-total-se-elaborar%C3%A1n-1-mill%C3%B3n-de-kits-covid-19-que-ser%C3%A1n-entregados-a-paciente/658779248327491/?locale=es_LA (accessed 3.15.23).

Hernández, A.L., 2021. Desautoriza el gobierno federal “tratamientos” de la Secretaría de Salud de CDMX contra Covid-19 [WWW Document]. Latinus. URL https://latinus.us/2021/08/03/desautoriza-gobierno-federal-tratamientos-secretaria-salud-cdmx-contra-covid-19/ (accessed 4.7.23).

Hernández Mares, P., 2021. Organizaciones médicas mexicanas rechazan uso de ivermectina y azitromicina para COVID-19 [WWW Document]. Medscape. URL https://espanol.medscape.com/verarticulo/5906516 (accessed 3.20.23).

IETSI, 2021. EsSalud actualiza sus tratamientos para los hospitalizados por covid-19 [WWW Document]. IETSI. URL https://ietsi.essalud.gob.pe/essalud-actualiza-sus-tratamientos-para-los-hospitalizados-por-covid-19/ (accessed 4.6.23).

IETSI, 2020. Uso de ivermectina para el tratamiento de pacientes con COVID-19 (8 de mayo de 2020).

IETSI, n.d. Nosotros – IETSI [WWW Document]. Instituto de Evaluación de Tecnologías en Salud e Investigación. URL https://ietsi.essalud.gob.pe/nosotros/ (accessed 4.6.23).

Instituto de Evaluación Tecnológica en Salud, 2020. Boletín de prensa sobre ivermectina.

Invima, 2020. Invima en Twitter: "La Ivermectina fue retirada del estudio clínico #Solidaridad por no demostrar seguridad y eficacia en el uso de este medicamento en pacientes con COVID 19. Twitter.

Invima, n.d. Cuidado con el uso de la Ivermectina para tratar COVID-19 [WWW Document]. Invima. URL https://www.invima.gov.co/cuidado-con-el-uso-de-la-ivermectina-para-tratar-covid-19 (accessed 3.31.23).

Krauze, L., 2022. La decisión del gobierno de Claudia Sheinbaum de distribuir ivermectina marca un nuevo fondo en el mal manejo de la pandemia [WWW Document]. Washington Post. URL https://www.washingtonpost.com/es/post-opinion/2022/02/09/ivermectina-merino-claudia-sheinbaum-cdmx-pandemia-covid-19/ (accessed 5.2.23).

La República, 2021. Colegio Médico del Perú rechaza el uso de ivermectina para casos COVID-19 [WWW Document]. La República. URL https://larepublica.pe/sociedad/2021/03/23/colegio-medico-del-peru-no-recomienda-uso-de-ivermectina-para-casos-covid-19 (accessed 4.6.23).

Larios, B., 2022. Proam ha entregado más de medio millón de kits de medicamentos contra COVID-19 [WWW Document]. Agencia Guatemalteca de Noticias. URL https://agn.gt/proam-ha-entregado-mas-de-medio-millon-de-kits-de-medicamentos-contra-el-covid-19/ (accessed 3.15.23).

Latinus, 2021a. Pese a que OMS aún analiza la ivermectina; IMSS seguirá usando el fármaco en pacientes con Covid-19 [WWW Document]. Latinus. URL https://latinus.us/2021/02/03/imss-seguira-suministrando-ivermectina-para-tratar-covid-19-zoe-robledo/ (accessed 10.31.23).

Latinus, 2021b. “López-Gatell es vocero; yo estoy en la acción más que en la promoción”, afirma Alcocer [WWW Document]. Latinus. URL https://latinus.us/2021/10/26/lopez-gatell-vocero-visible-accion-promocion-alcocer-comparecencia/ (accessed 4.6.23).

Le Lous, F., 2021. Ivermectina: ¿funciona contra la covid-19 un antiparasitario usado en vacas, caballos y perros? [WWW Document]. La Nación. URL https://www.nacion.com/blogs/el-explicador/ivermectina-funciona-contra-la-covid-19-un/GY5JEBCLXBEHJF6RQWVVOGGERU/story/ (accessed 3.15.23).

López, V., 2021. Aunque la ANMAT todavía no lo aprobó, se extiende el uso de la Ivermectina para tratar casos de coronavirus [WWW Document]. Clarín. URL https://www.clarin.com/sociedad/anmat-todavia-aprobo-extiende-uso-ivermectina-tratar-casos-coronavirus_0_qjoSpLG-F.html (accessed 4.12.23).

López-Medina, E., López, P., Hurtado, I.C., Dávalos, D.M., Ramirez, O., Martínez, E., Díazgranados, J.A., Oñate, J.M., Chavarriaga, H., Herrera, S., Parra, B., Libreros, G., Jaramillo, R., Avendaño, A.C., Toro, D.F., Torres, M., Lesmes, M.C., Rios, C.A., Caicedo, I., 2021. Effect of Ivermectin on Time to Resolution of Symptoms Among Adults With Mild COVID-19: A Randomized Clinical Trial. JAMA 325, 1426–1435. https://doi.org/10.1001/JAMA.2021.3071

Luna, C., 2020. Repartirán 10 mil kits con Ivermectina para combatir Covid-19 en Tuxtla [WWW Document]. El Sie7e. URL https://www.sie7edechiapas.com/post/repartir%C3%A1n-10-mil-kits-con-ivermectina-para-combatir-covid-19-en-tuxtla (accessed 10.30.23).

Maritano, A.P., 2020. Colombia – Desaconsejan la Ivermectina como tratamiento para covid-19 [WWW Document]. Diario Jurídico. URL https://www.diariojuridico.com/colombia-desaconsejan-la-ivermectina-como-tratamiento-para-covid-19/ (accessed 3.31.23).

Mendoza, M., 2021. Renuncia la ministra de Salud de Guatemala, Amelia Flores [WWW Document]. CNN Español. URL https://cnnespanol.cnn.com/2021/09/15/renuncia-la-ministra-de-salud-de-guatemala-orix/ (accessed 3.14.23).

Ministerio de Salud, 2023. Dirección General de Medicamentos, Insumos y Drogas (DIGEMID) [WWW Document]. gob.pe. URL https://www.gob.pe/21087-ministerio-de-salud-direccion-general-de-medicamentos-insumos-y-drogas-digemid (accessed 10.30.23).

Ministerio de Salud, 2022. Regulación de la Salud [WWW Document]. Ministerio de Salud. URL https://www.ministeriodesalud.go.cr/index.php/regulacion-de-la-salud (accessed 10.31.23).

Ministerio de Salud, 2020a. Un comité de expertos evalúa el comportamiento del covid-19 en el país [WWW Document]. Ministerio de Salud. URL https://www.minsalud.gov.co/Paginas/Un-comite-de-expertos-evalua-el-comportamiento-del-covid-19-en-el-pais.aspx (accessed 10.31.23).

Ministerio de Salud, 2020b. Digemid valida fórmula estandarizada de preparado Ivermectina con asistencia de especialistas en farmacotecnia [WWW Document]. gob.pe. URL https://www.gob.pe/institucion/minsa/noticias/187712-digemid-valida-formula-estandarizada-de-preparado-ivermectina-con-asistencia-de-especialistas-en-farmacotecnia (accessed 4.6.23).

Ministerio de Salud Pública y Asistencia Social, n.d. Kit de Medicamentos para COVID-19.

Ministerio de Salud y Protección Social, 2020. Gobierno Nacional no recomienda la ivermectina como tratamiento para covid-19, Boletín de Prensa.

Morales, J., 2021. Ex ministra de Salud, Amelia Flores destaca lucha contra COVID-19 en informe de labores de su gestión [WWW Document]. Agencia Guatemalteca de Noticias. URL https://agn.gt/ex-ministra-de-salud-amelia-flores-destaca-lucha-contra-covid-19-en-informe-de-labores-de-su-gestion/ (accessed 3.15.23).

Moreno, P., 2021. La vacuna será solución a largo plazo y Sagasti no quiere usar ivermectina para mitigar catástrofe [WWW Document]. El Perfil. URL https://elperfil.pe/politica/la-vacuna-sera-solucion-a-largo-plazo-y-sagasti-no-quiere-usar-ivermectina-para-mitigar-catastrofe/ (accessed 5.2.23).

Ola, A.L., 2020. Coronavirus: Criterios médicos no aprueban la distribución de kit de medicamentos [WWW Document]. Prensa Libre. URL https://www.prensalibre.com/guatemala/comunitario/coronavirus-criterios-medicos-no-aprueban-la-distribucion-de-kit-de-medicamentos/ (accessed 3.15.23).

Organización Panamericana de la Salud, 2020. Ongoing Living Update of Potential COVID-19 Therapeutics: summary of rapid systematic reviews (June).

Ramírez Coronel, M., 2022. Reparto de Ivermectina en la CDMX, desprestigio y falsa evidencia [WWW Document]. El Economista. URL https://www.eleconomista.com.mx/opinion/Reparto-de-Ivermectina-en-la-CDMX-desprestigio-y-falsa-evidencia-20220207-0002.html (accessed 5.2.23).

Redacción Animal Político, 2022. Cofepris debe informar sobre uso de ivermectina para COVID: INAI [WWW Document]. Animal Político. URL https://www.animalpolitico.com/sociedad/cofepris-ivermectina-pacientes-covid-inai (accessed 4.6.23).

Redacción El Comercio, 2020. Ucayali: EsSalud se prepara para producir 6 mil frascos de ivermectina para tratar el coronavirus [WWW Document]. El Comercio. URL https://elcomercio.pe/peru/ucayali-essalud-se-prepara-para-producir-6-mil-frascos-de-ivermectina-para-tratar-el-coronavirus-nnpp-noticia/ (accessed 4.6.23).

Redacción El País, 2020. Ivermectina para covid-19: estudio en Cali no dejó resultados alentadores [WWW Document]. El País. URL https://www.elpais.com.co/cali/ivermectina-para-covid-19-estudio-en-no-dejo-resultados-alentadores.html (accessed 4.7.23).

Redacción El Universal, 2022a. “No hubo ningún experimento”, dice López-Gatell sobre la entrega de ivermectina a pacientes Covid en la CDMX. El Universal.

Redacción El Universal, 2022b. Renuncia secretario de consejo de salubridad [WWW Document]. El Universal. URL https://www.eluniversal.com.mx/nacion/renuncia-secretario-de-consejo-de-salubridad/ (accessed 3.22.23).

Redacción Perú21, 2021a. Pilar Mazzetti sobre ivermectina: Minsa no puede entregar fármacos sin acreditación internacional [WWW Document]. Perú21. URL https://peru21.pe/lima/pilar-mazzetti-sobre-ivermectina-minsa-no-puede-entregar-farmacos-sin-acreditacion-internacional-coronavirus-peru-covid-19-nndc-noticia/ (accessed 4.4.23).

Redacción Perú21, 2021b. Óscar Ugarte sobre la ivermectina: “La OMS dice que no tiene utilidad por lo tanto no vamos continuar" [WWW Document]. Perú21. URL https://peru21.pe/lima/covid-19-ministro-oscar-ugarte-sobre-ivermectina-uso-no-continuara-porque-la-oms-dice-que-no-tiene-utilidad-coronavirus-nndc-noticia/ (accessed 4.4.23).

Redacción RPP, 2021. Doctor Palacios Celi: Colegio Médico del Perú no avala el uso de la ivermectina [WWW Document]. RPP. URL https://rpp.pe/peru/actualidad/doctor-palacios-celi-colegio-medico-del-peru-no-avala-el-uso-de-la-ivermectina-noticia-1314567 (accessed 4.6.23).

Redacción RPP, 2020a. Coronavirus en Perú: Martín Vizcarra anunció adquisición de hidroxicloroquina e ivermectina para tratar la COVID-19 [WWW Document]. RPP. URL https://rpp.pe/politica/gobierno/coronavirus-en-peru-martin-vizcarra-anuncio-adquisicion-de-hidroxicloroquina-e-ivermectina-para-tratar-la-covid-19-noticia-1270878 (accessed 4.4.23).

Redacción RPP, 2020b. Martín Vizcarra sobre Patricia Pimentel: “No hay ciencia exacta para saber el tratamiento” [WWW Document]. RPP. URL https://rpp.pe/peru/actualidad/martin-vizcarra-patricia-pimentel-no-hay-ciencia-exacta-para-saber-el-tratamiento-fiorella-molinelli-noticia-1298047?ref=rpp (accessed 4.6.23).

Redacción Semana, 2020. Iván Duque se refirió al uso de la ivermectina para la covid-19 | Colombia hoy [WWW Document]. Semana. URL https://www.semana.com/nacion/articulo/ivan-duque-se-refirio-al-uso-de-la-ivermectina-para-la-covid-19--colombia-hoy/686110/ (accessed 3.31.23).

Redacción Vigilante, 2023. Con 11 ministros de Salud en 3 años se complica la implementación de reformas adecuadas en el sector - Vigilante [WWW Document]. Vigilante. URL https://vigilante.pe/2023/01/06/con-11-ministros-de-salud-en-3-anos-se-complico-la-implementacion-de-reformas-adecuadas-en-el-sector/ (accessed 10.31.23).

Rodríguez, I., 2022. Hidroxicloroquina, plasma convaleciente y otras terapias que ya no se usan contra covid-19 ¿por qué? [WWW Document]. La Nación. URL https://www.nacion.com/ciencia/salud/hidroxicloroquina-plasma-convaleciente-y-otras/7EPS2NN7PBGDXMY3DLZJWNJDT4/story/ (accessed 3.15.23).

Rodríguez, I., Ávalos Rodríquez, Á., 2022. ¿Cómo escoge la CCSS qué medicamentos dar a pacientes con covid-19? [WWW Document]. La Nación. URL https://www.nacion.com/ciencia/salud/como-escoge-la-ccss-que-medicamentos-dar-a/KCQZK572SZB4VPCRXXJOZRQJAI/story/ (accessed 3.15.23).

Román, J., Rivera, D., Dominguez Velasco, M.A., 2020. Salud busca que el kit ambulatorio contra el coronavirus evite saturación en hospitales y prepara evento para adquirirlos [WWW Document]. Prensa Libre. URL https://www.prensalibre.com/guatemala/politica/salud-busca-que-el-kit-ambulatorio-contra-el-coronavirus-evite-saturacion-en-hospitales-y-prepara-evento-para-adquirirlos/ (accessed 3.15.23).

Rosario3, 2021. Piden a la Anmat que responda sobre el uso de Ivermectina contra el covid [WWW Document]. Rosario3. URL https://www.rosario3.com/informaciongeneral/Piden-a-la-Anmat-que-responda-sobre-el-uso-de-Ivermectina-contra-el-covid-20210605-0029.html (accessed 4.12.23).

Salud con lupa, 2020. El Salvador, Guatemala y Bolivia ofrecen kits de medicinas para COVID-19 sin prever reacciones adversas [WWW Document]. Salud con lupa. URL https://saludconlupa.com/noticias/el-salvador-guatemala-y-bolivia-ofrecen-kits-de-medicinas-para-covid-19-sin-prever-reacciones-adversas/ (accessed 8.12.22).

Sánchez-Talanquer, M., González-Pier, E., Sepúlveda, J., Abascal-Miguel, L., Fieldhouse, J., del Río, C., Gallalee, S., 2020. La respuesta de México al Covid-19: Estudio de caso.

Santiago, D., 2021. La secretaria de Salud defiende plan contra COVID en la CDMX [WWW Document]. Expansión Política. URL https://politica.expansion.mx/cdmx/2021/11/16/en-el-congreso-cdmx-oliva-lopez-defiende-plan-anticovid-y-responde-a-oposicion (accessed 2.21.23).

Sarabia, D., 2022. CDMX gastó 29 mdp en tratamiento con ivermectina no autorizado contra COVID [WWW Document]. Animal Político. URL https://www.animalpolitico.com/salud/gobierno-cdmx-gasto-tratamiento-covid-ivermectina (accessed 2.21.23).

Soto-Becerra, P., Culquichicón, C., Hurtado-Roca, Y., Araujo-Castillo, R. V, Domingo Cueto, J., floor, E., Maria Lima, J., 2020. Real-world effectiveness of hydroxychloroquine, azithromycin, and ivermectin among hospitalized COVID-19 patients: results of a target trial emulation using observational data from a nationwide healthcare system in Peru. medRxiv 2020.10.06.20208066. https://doi.org/10.1101/2020.10.06.20208066

The Associated Press, 2022. Peru swears in Dina Boluarte as new President amid constitutional crisis : NPR [WWW Document]. NPR. URL https://www.npr.org/2022/12/07/1141307938/peru-president-dissolves-congress-pedro-castillo (accessed 4.6.23).

The Tico Times, 2021. Caja asks government to impose new Covid measures [WWW Document]. The Tico Times. URL https://ticotimes.net/2021/05/10/caja-asks-government-to-impose-new-covid-measures-2 (accessed 3.15.23).

Tinoco-Mora, Z., 2005. SELECCIÓN DE MEDICAMENTOS EN LA CCSS COMITÉ CENTRAL DE FARMACOTERAPIA. Fármacos 18, 1–2.

Torres, F., 2020. Réplica y despido: el segundo informe que generó la salida de la gerenta de investigación de EsSalud [WWW Document]. Salud con Lupa. URL https://saludconlupa.com/noticias/replica-y-despido-este-es-el-segundo-informe-que-genero-la-salida-de-la-gerenta-de-investigacion-de-essalud/ (accessed 4.6.23).

Trigo, M.S., Kurmanaev, A., León Cabrera, J.M., 2020. Coronavirus en América Latina: algunas autoridades respaldan tratamientos cuestionables [WWW Document]. The New York Times. URL https://www.nytimes.com/es/2020/07/23/espanol/america-latina/bolivia-cloro-coronavirus-ivermectina.html (accessed 4.4.23).

UN Sustainable Development Group, 2020. 5 reasons Costa Rica is winning plaudits for fighting COVID-19 [WWW Document]. UNSDG. URL https://unsdg.un.org/latest/blog/5-reasons-costa-rica-winning-plaudits-fighting-covid-19 (accessed 2.8.23).

Vet Market, 2021. La ivermectina aún no fue autorizada por la ANMAT pero cada vez la utilizan más [WWW Document]. Vet Market. URL https://vetmarketportal.com.ar/nota/1531/la-ivermectina-aun-no-fue-autorizada-por-la-anmat-pero-cada-vez-la-utilizan-mas (accessed 4.17.23).

1. ^*^Jorge Alcocer, Health Minister, took a secondary role in the response.(Latinus, 2021b) In his place, President Andres Manuel Lopez Obrador assigned Hugo Lopez Gatell, Undersecretary of Prevention and Health Promotion, as the person in charge of the Mexican response during the pandemic.(Fonseca, 2020)
