# Supplementary material for: COVID-19 kit distribution in six Latin American countries: bureaucratic autonomy, populism, and evidence-based policymaking
Source: Health Policy Plan. 2026 May 20;41(7):1165–78. doi: 10.1093/heapol/czag069 (PMC13481690; doi:10.1093/heapol/czag069)
Supplement: Supplementary file 1 [file czag069_supplementary_data.zip › Table1.docx]

Table 1: COVID-19 kit content in five Latin American countries

|  | **El Salvador** | **Guatemala*** | **Brazil** | **Mexico*** | **Peru*** |
| --- | --- | --- | --- | --- | --- |
| **Acetaminophen** | **x** | **x** |  |  | **x** |
| **Acetylsalicylic acid** | **x** | **x** | **x** |  |  |
| **Azithromycin** | **x** | **x** |  | **x** | **x** |
| **Hydroxychloroquine** |  |  | **x** |  |  |
| **Ivermectin** | **x** | **x** | **x** | **x** | **x** |
| **Loratadine** | **x** | **x** |  |  |  |
| **Prednisone** |  |  | **x** |  |  |
| **Vitamin C, D and Zinc** | **x** | **x** |  |  |  |

*Countries included in this study
