# Supplementary material for: COVID-19 kit distribution in six Latin American countries: bureaucratic autonomy, populism, and evidence-based policymaking
Source: Health Policy Plan. 2026 May 20;41(7):1165–78. doi: 10.1093/heapol/czag069 (PMC13481690; doi:10.1093/heapol/czag069)
Supplement: Supplementary file 1 [file czag069_supplementary_data.zip › Table2.docx]

**Table 2: National Medicines Regulatory Agency (NMRA) budget allocation and authority to appoint agency leader.**

| Country | National Medicines Regulatory Agency | Part of the Ministry of Health | Who appoints the head of the agency? | Is the budget independent of the Ministry of Health? | Degree of autonomy |
| --- | --- | --- | --- | --- | --- |
| Argentina* | ANMAT - National Administration of Drugs, Food and Medical Technology | No | President of the Republic | Yes. The National Budget Office of the Ministry of Finance publishes detailed information regarding the budget allocated by law and budget execution. | Maximum (3) |
| Colombia* | Invima - National Institute for the Surveillance of Drugs and Food | No | President of the Republic | Yes. Resources allocated from the National budget. | Maximum (3) |
| Mexico | COFEPRIS - Federal Commission for Protection against Health Risks | Yes | President of the Republic instructs the Secretary of Health for appointment | No. | Medium (2) |
| Costa Rica | Department of Regulation of Products of Sanitary Interest (DRPIS) - Ministerio de Salud, **Regulación de Productos de Interés Sanitario**  Department of Pharmacoepidemiology** | Yes | Minister of Health  Director of the Costa Rican Social Security Fund (CCSS)** | No. | Medium (2) |
| Guatemala | Department of Regulation and Control of Pharmaceutical Products | Yes | Appointed by competent authorities of the Ministry of Public Health and Social Assistance (MSPAS) | No. Resources allocated from the federal government budget to the Ministry of Public Health and Social Assistance, who assigns a part to the Department. | Minimum (1) |
| Peru | DIGEMID- General Directorate of Medicines, Supplies and Drugs | Yes | Director General appointed by the Ministry of Health | No. | Minimum (1) |

Legend:

* Indicates that based on the assessment, the NMRAs were considered to have a high degree of autonomy;

** The Pharmacoepidemiology Department within Caja contributes to the autonomy score for Costa Rica because its head is appointed by the Director of the CCSS, an institution separate from the Ministry of Health.
