# Supplementary material for: COVID-19 kit distribution in six Latin American countries: bureaucratic autonomy, populism, and evidence-based policymaking
Source: Health Policy Plan. 2026 May 20;41(7):1165–78. doi: 10.1093/heapol/czag069 (PMC13481690; doi:10.1093/heapol/czag069)
Supplement: Supplementary file 1 [file czag069_supplementary_data.zip › Table3.docx]

**Table 3: Degrees of populist rhetoric of the political leader in the six countries studied.**

| Country | Political Party ^[1]^ | President | Administration Period | Populist Rhetoric Score  (0-2) | Executive strength in legislature | Left/right ideology (-1/1) |
| --- | --- | --- | --- | --- | --- | --- |
| Guatemala | Vamos | Alejandro Giammattei | January 2020-January 2024 | 0.16 | 0.11 | Right (1) |
| Argentina | Partido Justicialista / Frente de Todos | Alberto Fernández | December 2019 – December 2023 | 0.29 | 0.47 | Left (-1) |
| Mexico | MORENA | Andrés Manuel López Obrador | December 2018 – October 2024 | 0.96 | 0.63 | Left (-1) |
| Colombia | Partido Centro Democrático | Iván Duque | August 2018 – August 2022 | 0 | 0.20 | Right (1) |
| Peru | Not affiliated with a party^2^ | Martín Vizcarra | March 2018 - November 2020 | 0.14 | 0 | Right (1) |
| Costa Rica | Partido Acción Ciudadana (PAC) | Carlos Alvarado Quesada | May 2018 – May 2022 | 0 | 0.18 | Left (-1) |
| Region: median |  |  |  | 0.19 |  |  |

Legend:

Political party of the president at the time of the pandemic (from December 2019 to August 2022)

^2^ President Vizcarra originally served as Vice President of Pedro Pablo Kuczynski’s administration, affiliated with *Peruanos por el Kambio*, but post-2019 was not formally affiliated with any party.

"Populist Rhetoric Score" is a scale from 0 to 2, where a higher score indicates stronger populist rhetoric.

“Executive strength in legislature” presents the percentage of seats held by the president’s party in the lower chamber of the legislature.

"The “Left/right ideology" column describes the ideological values associated with the president, from the GPD data; options Right, Centrist, and Left are coded from -1 (left) to 1 (right).

1. Political Parties in power at the time of the pandemic (from December 2019 to August 2022)

   "Populist Rhetoric Index" is a scale from 0 to 10, where a higher score indicates a stronger populist rhetoric.

   "Type of Populism" column categorises the populism as either “Moderately Populist”, "Strongly Populist", "Moderately Pluralist", or “Strongly Pluralist.”

   "Political Values" column describes the ideological values associated with the respective parties and being Right Conservative, Left Liberal, Right Liberal and Left Conservative.
