# Supplementary material for: COVID-19 kit distribution in six Latin American countries: bureaucratic autonomy, populism, and evidence-based policymaking
Source: Health Policy Plan. 2026 May 20;41(7):1165–78. doi: 10.1093/heapol/czag069 (PMC13481690; doi:10.1093/heapol/czag069)
Supplement: Supplementary file 1 [file czag069_supplementary_data.zip › Table4.docx]

**Table·4: Overview of National and Local Stakeholders.**

| Category | Guatemala | Costa Rica | Mexico | Colombia | Peru | Argentina |
| --- | --- | --- | --- | --- | --- | --- |
| President | Alejandro Giammattei | Carlos Alvarado | Andrés Manuel López Obrador | Ivan Duque | Martín Vizcarra | Alberto Fernández |
| Health Minister | Amelia Flores  Francisco Coma Martin | Daniel Salas | Oliva López Arellano* | Fernando Ruiz Gómez | Victor Zamora | Ginés González García |
| Director of Social Security/ Vice Minister | Not specified | Roman Macaya | Zoé Robledo | Luis Alexander Moscoso | Fiorella Molinelli | Not specified |
| Medical Associations | GAID^1^ GIMA^2^  CMAG^3^  CPSG^4^ | Not specified | Mexican Associations of Infectious Diseases and Clinical Microbiology (MAIDCM)  Mexican College of Critical Medicine (MCCM) | Colombian Association of Infectious Diseases (ACIN) | Medical College of Peru | Argentine Society of Infectious Diseases (SADI) |
| Public Healthcare Provider | Ministry of Public Health and Social Assistance (MPHSA) | Caja Costarricense de Seguro Social (CCSS) | Mexican Institute of Social Security (IMSS) | Ministry of Health and Social Protection (MHSP)  Health Promoting Entities (EPS)  Health Service Provider Institutions (IPSS) | Minsa/EsSalud | Ministerio de Salud Argentina |
| National Regulatory Agency | DRCPHP^5^ | DRPIS^6^  Pharmaco-epidemiology Directorate (Pharm) | Federal Commission for the Protection Against Sanitary Risks (COFEPRIS) | National Institute for Food and Drug Surveillance (Invima) | General Directorate of Medicines, Supplies, and Drugs (DIGEMID) | National Administration of Drugs, Food, and Medical Technology (ANMAT) |
| Health Technology Assessment Organizations | Not specified | National Center for Biotechnological Innovations | National Center for Technological Excellence in Health (CENETEC) | Institute for Technological Evaluation of Health (IETS) | CONCYTEC^7^  IETSI^8^ | National Commission for Evaluation of Health Technologies (CONETEC) |

*Local Health Minister (Mexico City)

^1^ Guatemala Association of Infectious Diseases

^2^ Guatemalan Internal Medicine Association

^3^ Critical Medicine Association of Guatemala

^4^ College of Physicians and Surgeons of Guatemala

^5^ Department of Regulation and Control of Pharmaceutical and Related Products

^6^ Department of Regulation of Products of Sanitary Interest

^7^ National Council of Science, Technology, and Technological Innovation

^8^ Institute for Health Technology Assessment and Research
